# Supplementary material for: The master male sex determinant Gdf6Y of the turquoise killifish arose through allelic neofunctionalization
Source: Nat Commun. 2025 Jan 9;16:540. doi: 10.1038/s41467-025-55899-7 (PMC11718055; doi:10.1038/s41467-025-55899-7)
Supplement: Supplementary file 1 — Supplementary Information [file 41467_2025_55899_MOESM1_ESM.pdf]

SUPPLEMENTARY INFORMATION

The master male sex determinant Gdf6Y of the turquoise killifish arose through allelic neofunctionalization

Annekatrin Richter,[1,*] Hanna Mörl,[1] Maria Thielemann,[1,4] Markus Kleemann,[1,5] Raphael Geißen,[1,6] Robert Schwarz,[1] Carolin Albertz,[1] Philipp Koch,[1] Andreas Petzold,[1,7] Torsten Kroll,[1] Marco Groth,[1] Nils Hartmann,[1,8] Amaury Herpin,[2] Christoph Englert[1,3,*]

[1]Leibniz Institute on Aging - Fritz Lipmann Institute (FLI), Beutenbergstraße 11, 07745 Jena, Germany

[2]INRAE - LPGP, 35000 Rennes, France

[3]Institute of Biochemistry and Biophysics, Friedrich Schiller University Jena, Hans-Knöll-Straße 2, 07745 Jena, Germany

[4]Present address: BianoGMP GmbH, Ronneburger Straße 74, 07546 Gera, Germany

[5]Present address: Abbott Rapid Diagnostics Jena GmbH, Orlaweg 1, 07743 Jena, Germany

[6]Present address: Memorial Sloan Kettering Cancer Center, 1275 York Ave, New York, NY 10065, USA

[7]Present address: DRESDEN-concept e. V., Technical University (TU) Dresden, 01062 Dresden, Germany

[8]Present address: Institute of Pathology, University Medical Center of the Johannes Gutenberg University Mainz, Langenbeckstraße 11, 55131 Mainz, Germany

*Corresponding authors: Annekatrin.Richter@leibniz-fli.de
Christoph.Englert@leibniz-fli.de

SUPPLEMENTARY FIG. 1

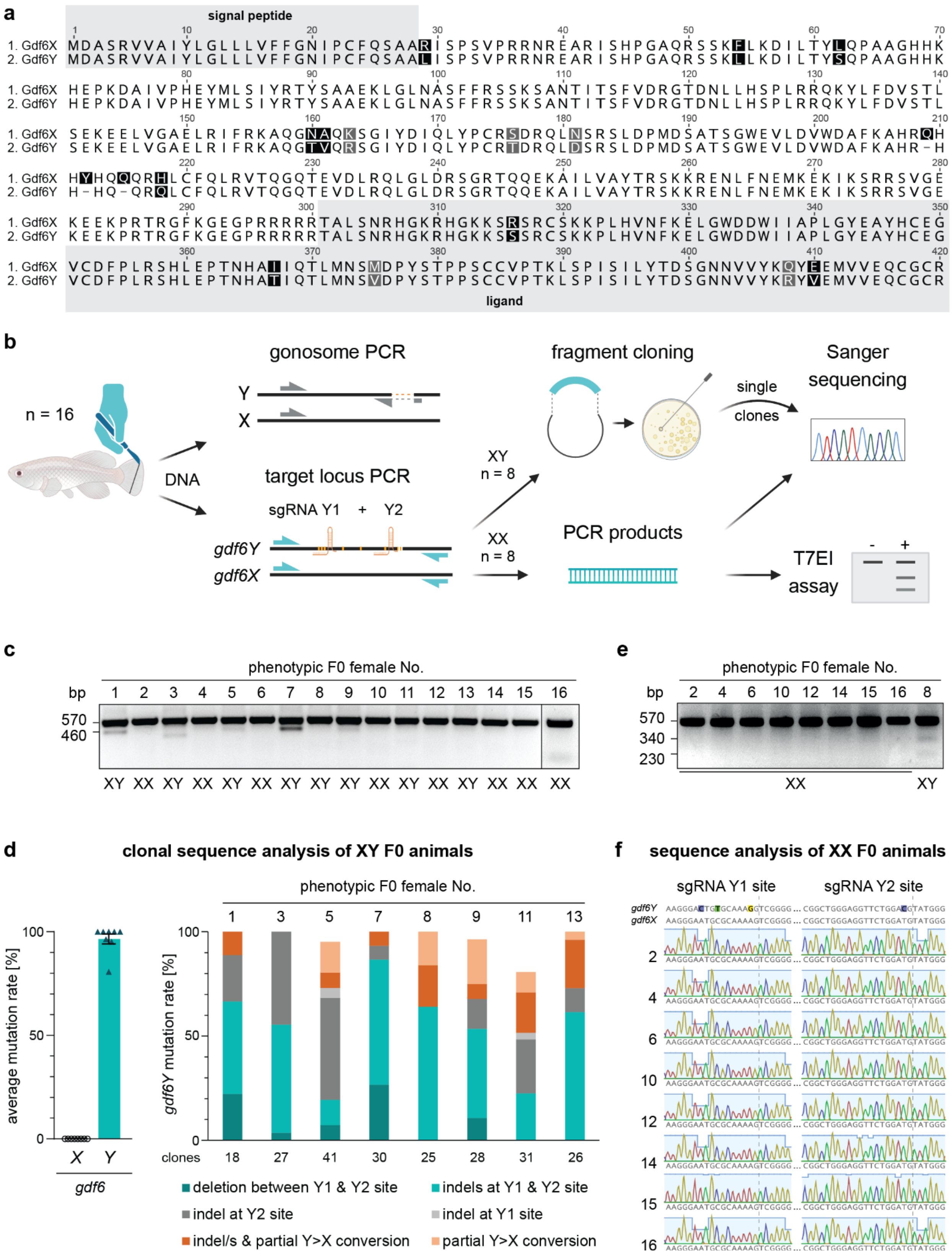

Supplementary Fig. 1 | Comparison of Gdf6X and Gdf6Y and analyses of mosaic F0 *gdf6Y* mutants. a Alignment of 1. Gdf6X and 2. Gdf6Y amino acid sequences (one-letter code). Sequence differences are highlighted in gray for similar and black for dissimilar amino acids. **b** Schematic of *gdf6Y* F0 mutant analysis. T7EI – T7 endonuclease I. Created in BioRender. Richter, A. (2024) https://BioRender.com/o66n598. **c** Ethidium bromide-stained agarose gel picture of the sgRNA Y1 and Y2 target locus PCR on fin tissue lysates from the 16 phenotypically female CRISPR/Cas9-mutated F0 animals. The sex chromosomes of each individual, as derived from the gonosome PCR, are indicated below the samples. **d** Result of the clonal sequence analysis of the 8 CRISPR/Cas9-mutated XY F0 animals. Left: Average mutation rates in *gdf6X* and *gdf6Y* with the standard error of the mean (SEM) and individual values. Right: *Gdf6Y* mutation rates in the individual animal samples are separated into the different observed mutation types. Apart from insertion and deletion (indel) events at either or both sgRNA target sites, partial gene conversion between *gdf6X* and *gdf6Y* was observed probably due to homology-directed repair of DNA double-strand breaks. **e** Ethidium bromide-stained agarose gel picture of the T7EI assay on target locus PCR products from the 8 CRISPR/Cas9-mutated XX F0 animals. No mutations were detected compared to the CRISPR/Cas9-mutated XY F0 animal sample. **f** Partial sequence alignments with chromatograms of target locus PCR products from the 8 CRISPR/Cas9-mutated XX F0 animals. No double peaks or decreased sequence qualities (light blue columns) were detected in chromatograms at the putative Cas9 cleavage sites mediated by sgRNA Y1 and Y2 (dashed lines). **c–e** Source data are provided as a Source Data file.

SUPPLEMENTARY FIG. 2

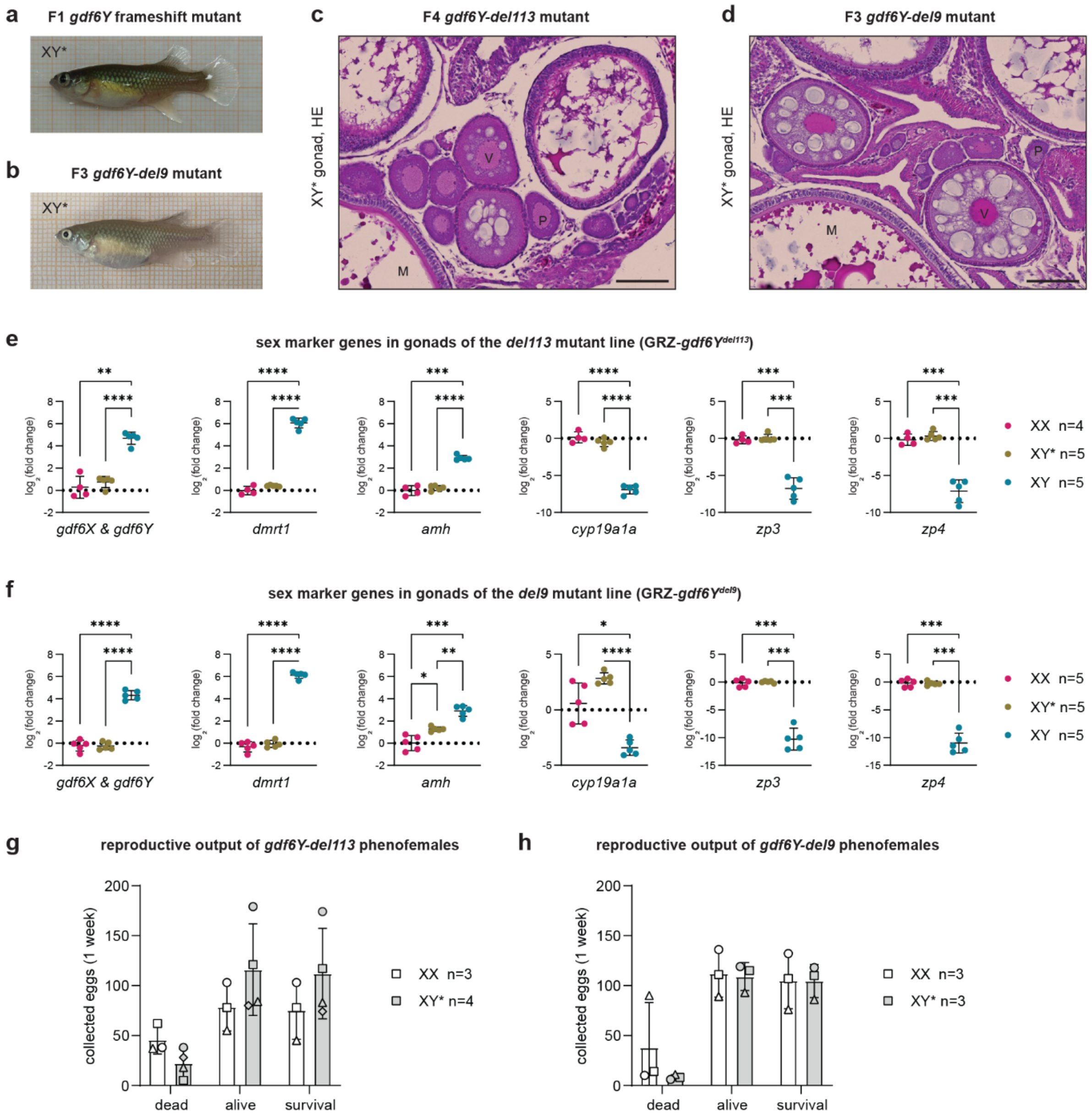

Supplementary Fig. 2 | The phenofemale phenotype and fertility in subsequent generations. Phenotypes of (**a**) an F1 phenofemale (XY*) with a frameshift causing indel (*del4, ins3*) in *gdf6Y* and (**b**) an F3 GRZ-$gdf6Y^{del9}$ phenofemale at 2.4 and 1.5 months of age, respectively. HE stained gonadal sections of (**c**) an F4 GRZ-$gdf6Y^{del113}$ phenofemale and (**d**) an F3 GRZ-$gdf6Y^{del9}$ at about 3 months of age. Expression of *gdf6Y* together with *gdf6X* as well as male and female marker genes on mRNA-level in (**e**) F4 GRZ-$gdf6Y^{del113}$ and (**f**) F3 GRZ-$gdf6Y^{del9}$ XX females, XY* phenofemales, and XY males at about 3 months of age (mean with standard deviation). Statistical testing by Welch's ANOVA and Dunnett's T3 multiple comparisons test (others), (*) $P<0.05$, (**) $P<0.01$, (***) $P<0.001$, (****) $P<0.0001$. Numbers of dead or alive eggs collected after 1 week of constant breeding of single males with single phenofemales from (**g**) the F7 generation of GRZ-$gdf6Y^{del113}$ or (**h**) the F5 generation of GRZ-$gdf6Y^{del9}$ at about 3 months of age and the numbers of surviving eggs after 1 week of incubation at 25°C (mean with standard deviation). Stratified statistical testing between XX and XY* by two-tailed Welch's t-tests. **e–h** Source data are provided as a Source Data file.

SUPPLEMENTARY FIG. 3

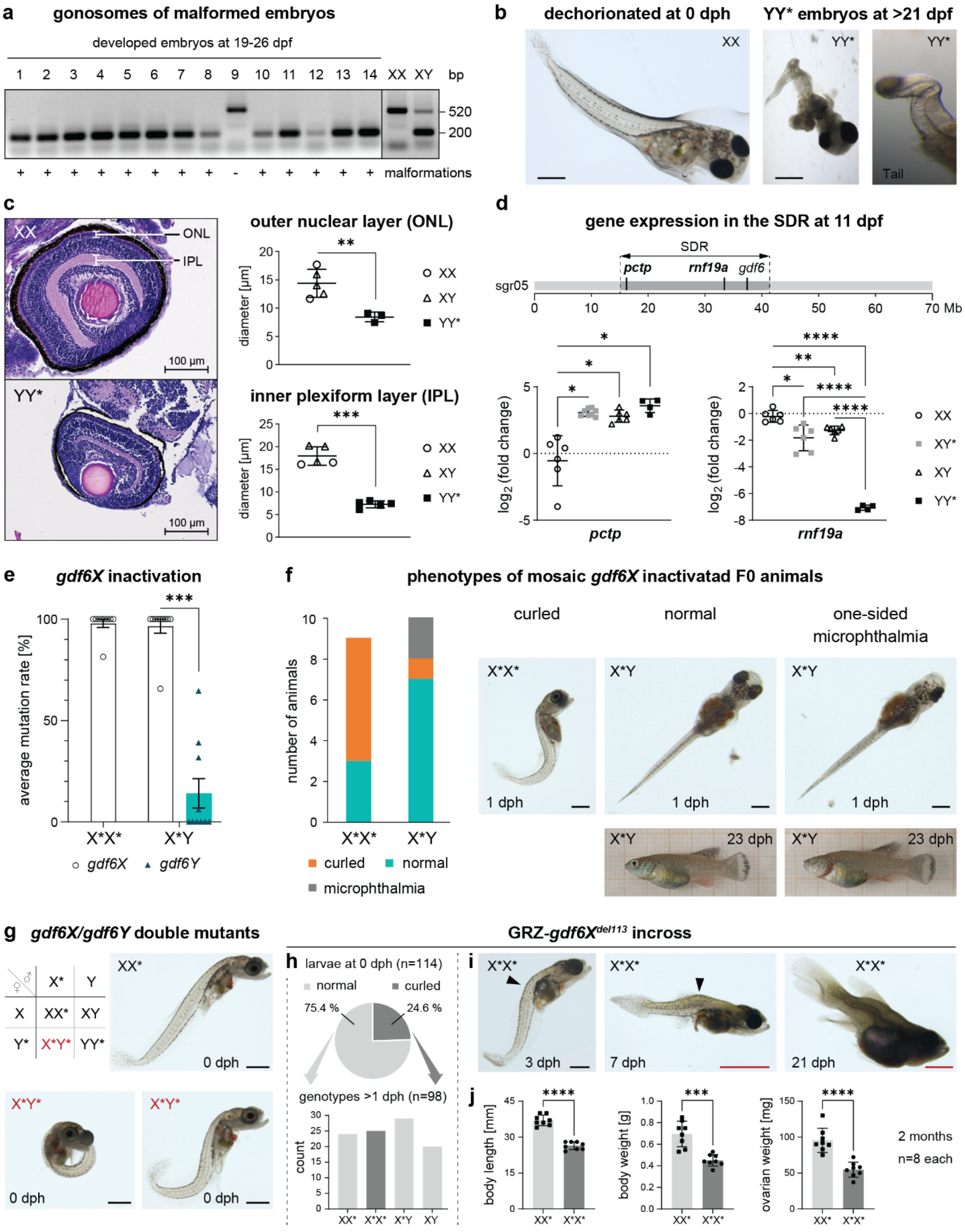

Supplementary Fig. 3 | YY* embryo and *gdf6X* mutant phenotypes. **a** Ethidium bromide-stained agarose gel picture of molecular sexing utilizing an amplicon length polymorphism between gonosomes. Malformed embryos have only Y-chromosomes indicating them as YY* embryos (GRZ-*gdf6Y*$^{del6,\ del8}$ from Fig. 1i). **b** Dechorionated YY* embryos (GRZ-*gdf6Y*^{del113}) have unpigmented, malformed tails, and smaller eyes than normally developing animals (XX) at 0 dph. **c** Left: HE stained normally developed (XX) and YY* (GRZ-*gdf6Y*$^{del6,\ del8}$) eyes at 0 dph or 19-26 dpf, respectively. Right: Diameter quantification of the retinal outer nuclear (ONL) and inner plexiform layer (IPL) of normally developed (XX, n=2; XY, n=3) and YY* (GRZ-*gdf6Y*$^{del6,\ del8}$; ONL, n=3; IPL, n=5) animals. Average layer diameter measurements at three positions per sample using ZEN 2.6 (Blue Edition, Zeiss). Statistical testing by a two-tailed Welch's *t*-test. **d** Chromosomal location and expression of *pctp* and *rnf19a* in trunks of XX (n=6), XY* (GRZ-*gdf6Y*^{del9}; *pctp*: n=8, *rnf19a1a*: n=6), XY (n=6), and YY* (n=4) embryos at 11 dpf. SDR – sex-determining region. Sgr05 – synteny group 5. Statistical testing by Welch's ANOVA and Dunnett's T3 multiple comparisons test. **e** Result of sequence analyses with the Synthego ICE tool (v3.0) of 19 *gdf6X*-mosaic F0 animals as average mutation rate with SEM. Statistical testing within the X*Y group by a one-tailed Wilcoxon matched-pairs signed rank test. **f** Genotypes and phenotypes of *gdf6X*-mosaic F0 animals. Predominantly X*X* animals had a curled phenotype. One-sided microphthalmia was male-specific (X*Y). **g** Dechorionated *gdf6X*$^{-}$/*gdf6Y*^{del9} double mutants at 0 dph (X*Y*; n=30) have the curled phenotype (two manifestations shown) compared to developed siblings (XX*, n=30; XY, n=29). **h** Phenotype and genotype proportions among the offspring of a GRZ-*gdf6X*^{del113} F2 incross. **i** Phenotype of homozygous F3 *gdf6X*-mutants (X*X*) over time. Arrowhead: spine kink. **j** Body and organ parameters of X*X* and XX* animals. Statistical testing by two-tailed Welch's *t*-tests. **b, f, g, h** Scale bars: Black, 500 µm; Red, 2 mm. **c, d, j** Mean with standard deviation. **c, d, e, j** (*) $P<0.05$, (**) $P<0.01$, (***) $P<0.001$, (****) $P<0.0001$. **a, c–f, h, j** Source data are provided as a Source Data file.

SUPPLEMENTARY FIG. 4

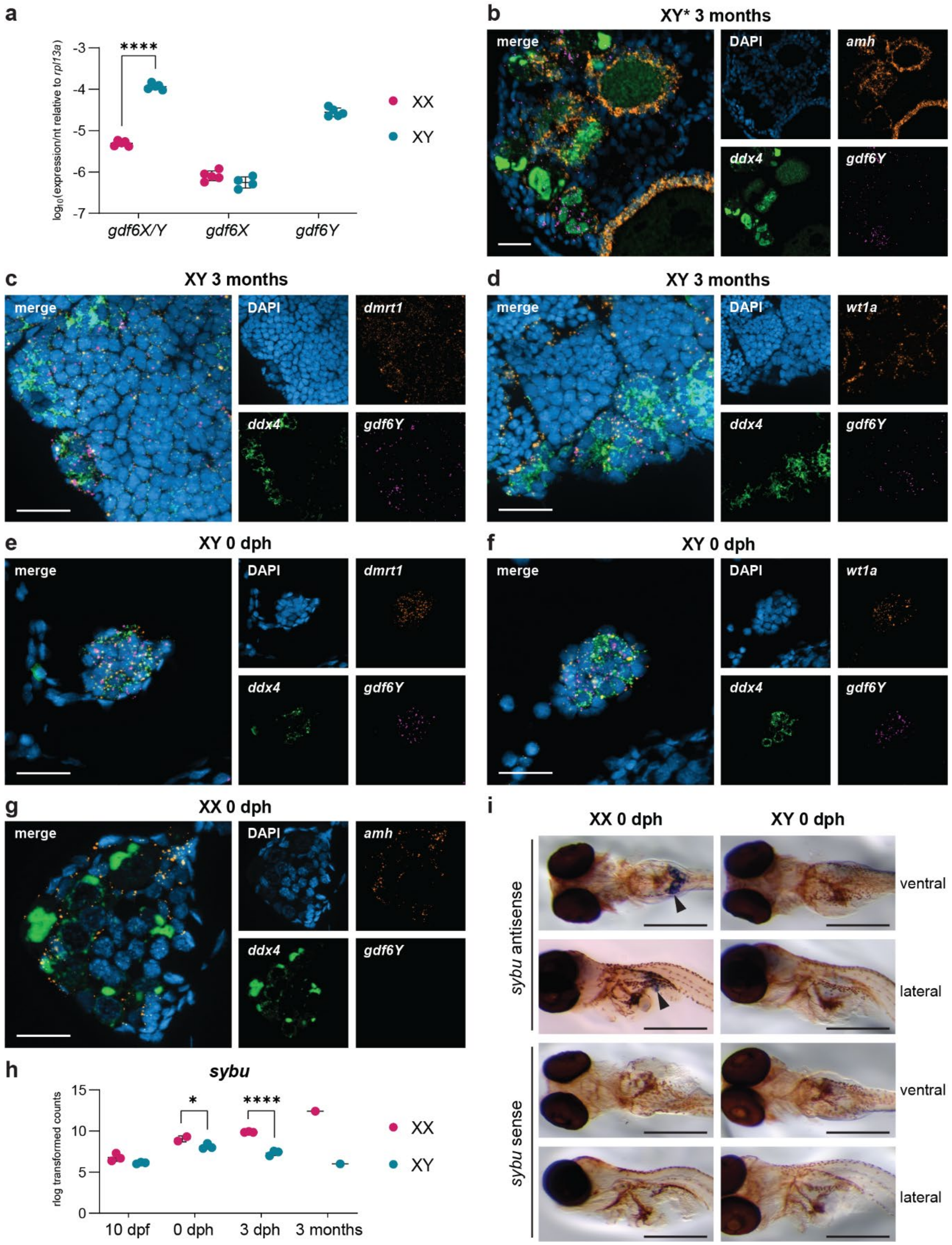

Supplementary Fig. 4 | *Gdf6Y* expression in *N. furzeri.* **a** Expression of *gdf6X* and *gdf6Y* together and separately relative to *rpl13a* in gonads of females (XX; n=5) and males (XY; *gdf6X*, n=4; *gdf6X/Y*, *gdf6Y*, n=5) at about 4 months of age. Statistical testing by stratified two-tailed Welch's *t*-tests for *gdf6X/Y* and *gdf6X*. Source data are provided as a Source Data file. **b–g** *Gdf6Y* (magenta) transcripts, the GC marker *ddx4* (green), and one somatic cell marker were detected. White scale bars: 20 µm. The somatic cell marker *amh* (orange) was detected in (**b**) a sexually mature phenofemale (n=5; here: GRZ-$gdf6Y^{del113}$, 3 months old) and (**g**) a female 0 dph ovary (n=5). The somatic cell markers *dmrt1* (**c, e**) and *wt1a* (**d, f**) were detected in (**c, d**) the testes of 3 months old (n=3) and (**e, f**) 0 dph animals (*dmrt1*, n=4; *wt1a*, n=6). **h** Expression analysis of *sybu* by RNA-Seq[1] in the whole embryo at 10 dpf (n=3 each), the trunk at 0 (XX, n=2; XY, n=3 each) or 3 dph (n=3 each), and a gonad at 3 months of age in XX and XY animals. Rlog-transformed counts with DESeq2 FDR (Supplementary Data 1). **a, h** (*) $P<0.05$, (****) $P<0.0001$. **i** Whole mount *in situ* hybridization of MZM0403 hatchlings with *sybu*-mRNA antisense (females: n=5, males: n=3) and sense probes (n=8; definitive females: n=3, definitive males: n=3). Black scale bars: 500 µm. Arrows: Oocytes stained with the antisense probe.

SUPPLEMENTARY FIG. 5

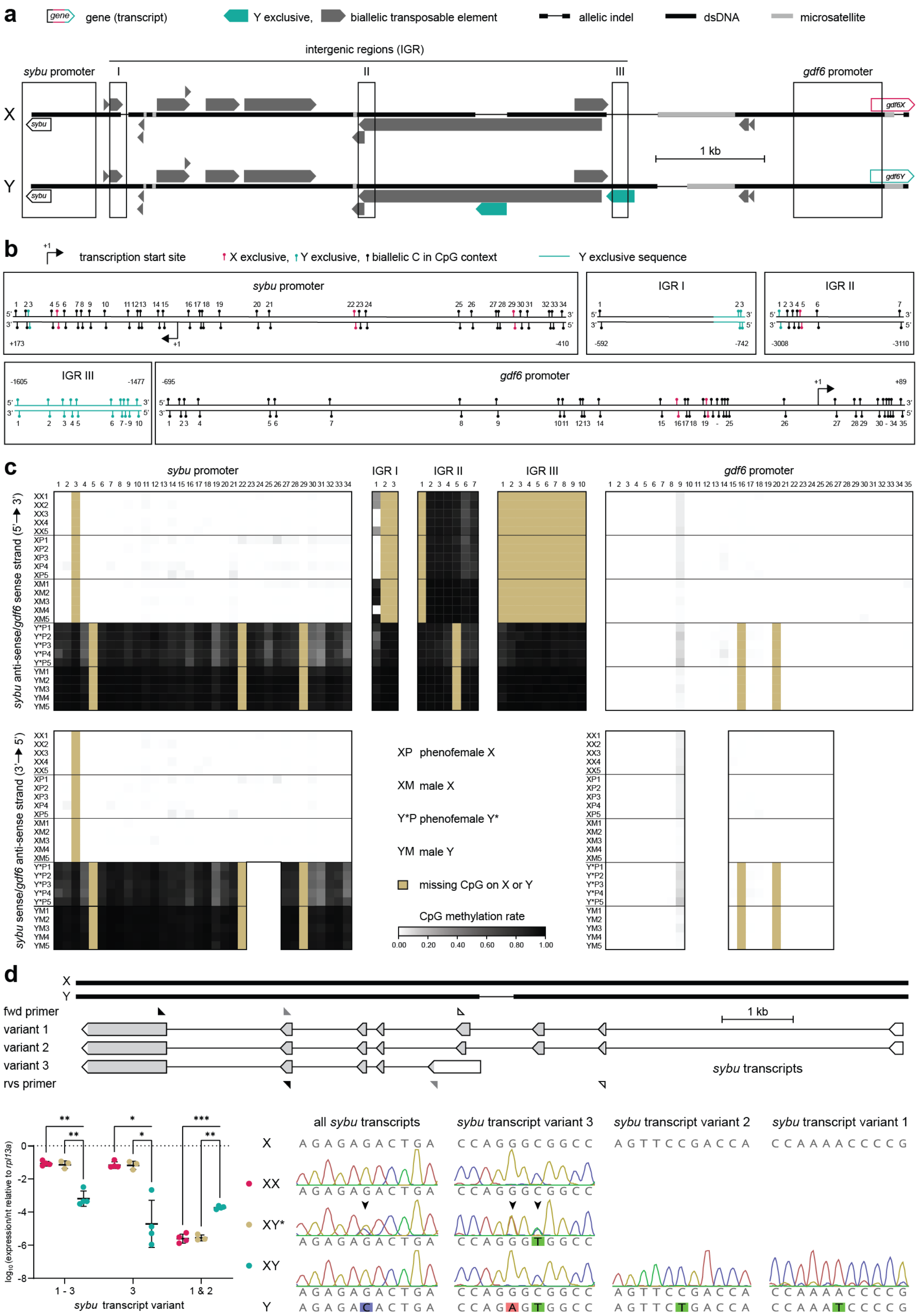

Supplementary Fig. 5 | DNA methylation of the genomic region between *sybu* and *gdf6X/Y* on the sex chromosomes in gonads. **a** Schematic of the genomic region of the X and Y chromosome containing Y exclusive TEs (*gdf6Y*-distal TE: NfRep007207 of unknown identity; *gdf6Y*-proximal TE: partial retrotransposon NfRep000041 of LINE/RTE-BovB type; source: https://nfingb.leibniz-fli.de). **b** The CpG positions of the analyzed subregions are shown. *(c)* Heatmaps of CpG methylation rates on the X and Y or Y* chromosomes in the F2 gonads of the analyzed 2 months old females (XX), phenofemales (P, GRZ-*gdf6Y*^{del113}), and males (M; n=5 each). For some locations, the *sybu* sense strand (bottom panels) was analyzed in addition to the *gdf6X/Y* sense strand (upper panels). *(d)* Schematic and sex-specific expression (mean with standard deviation) of three different *sybu* transcripts in the F1 gonads of 2.4 months old females (XX, n=4), phenofemales (Supplementary Table 1; XY*, n=3), and males (XY, n=4). Exons and transcript-specific primer pairs are indicated with triangles (black – transcript variants 1-3, gray – variant 3, white – variant 1 and 2). *Sybu* cDNA sequences indicate an X-exclusive transcription in males, while expression from the Y chromosome is reactivated in phenofemales. Stratified statistical testing by Welch's ANOVAs and Dunnett's T3 multiple comparisons tests per detected transcript variants, (*) $P<0.05$, (**) $P<0.01$, (***) $P<0.001$. **c, d** Source data are provided as a Source Data file.

SUPPLEMENTARY FIG. 6

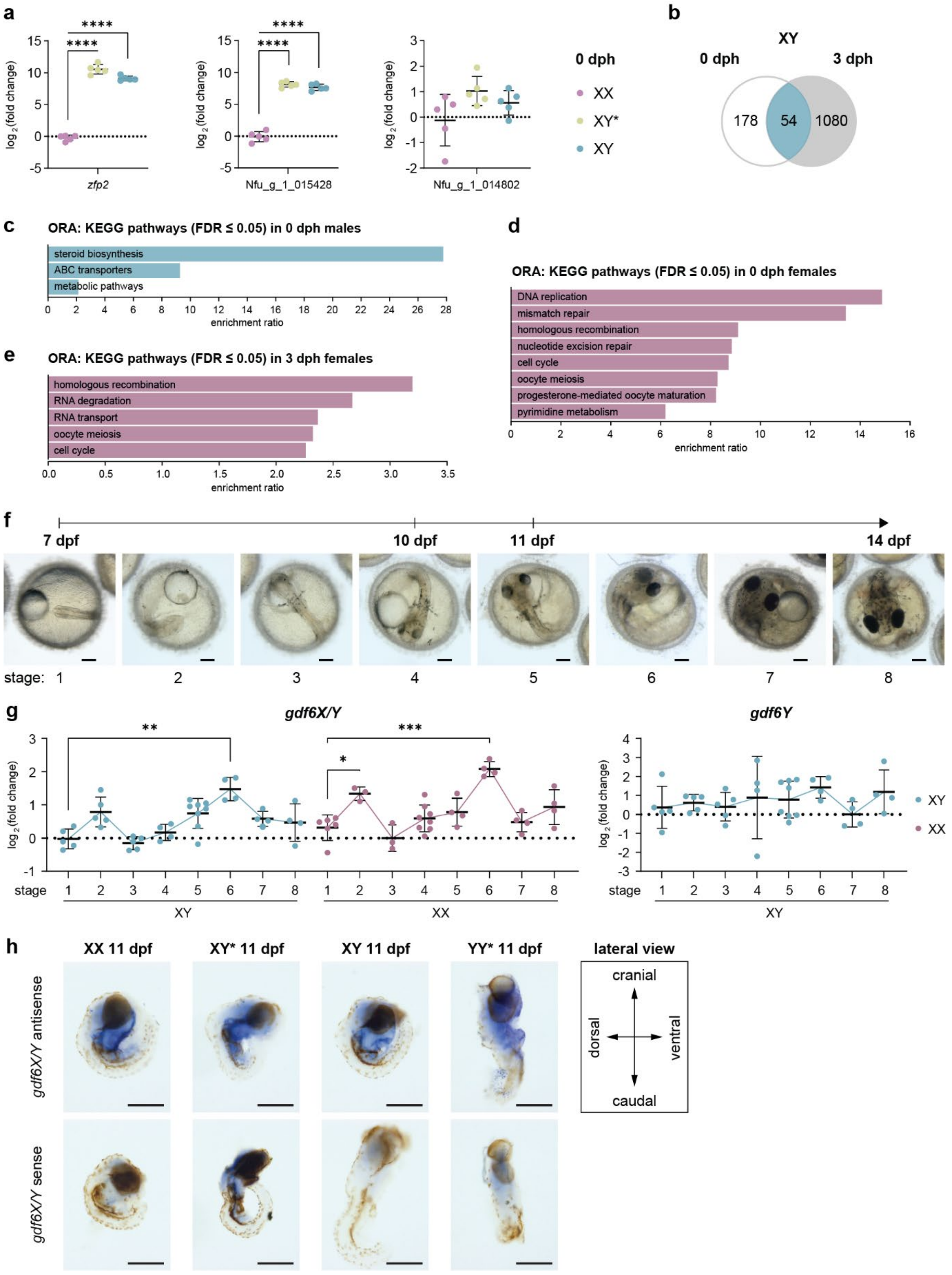

Supplementary Fig. 6 | RNA-Seq data[1] reveal that oogenesis starts before hatching, while male and female embryos express *gdf6X/Y* indistinguishably. a Expression of the top three DEGs at 10 dpf in trunks of XX, XY* (GRZ-$gdf6Y^{del113}$), and XY animals at 0 dph (n=5 each). Statistical testing by Welch's ANOVA and Dunnett's T3 multiple comparisons tests with XX. **b** Overlap of male DEGs between 0 and 3 dph. **c, d, e** Overrepresentation analysis (ORA) of KEGG pathways within the (**c**) male- and (**d**) female-specific DEGs at 0 dph and (**e**) the female-specific DEGs at 3 dph. **f** Embryonic stages observed between 7 and 14 dpf in eggs incubated in 0.3x Danieau's medium at 29°C to promote direct development without diapause. Stage 1 corresponds to the somite stage, in which diapause entry would occur, while organogenesis is completed at stage 8. Stages used in other experiments (10 and 11 dpf) are indicated. **g** RT-qPCR expression analyses of *gdf6X* and *gdf6Y* together (*gdf6X/Y*) and *gdf6Y* separately at embryonic stages from (**f**). Stages 1-5: RNA-isolated from whole embryos and PCR-sexing after cDNA syntheses (XY: stages 1-3, 4, and 5 with n=5 each, n=4, and n=8, respectively; XX: stages 1, 2-3, 4, and 5 with n=6, n=3 each, n=8, and n=4, respectively). Stages 6 to 7: PCR-sexing on tail tips and RNA-isolation from the remaining embryo. Stage 8: PCR-sexing on heads and tails and RNA-isolation from trunk parts (n=4 each). Statistical testing by Welch's ANOVA and Dunnett's T3 multiple comparisons tests between stage 1 and subsequent stages per sex, and between sexes of each stage if applicable. **a, g** (*) $P<0.05$, (**) $P<0.01$, (***) $P<0.001$, (****) $P<0.0001$. **h** Whole-mount *in situ* hybridization of yolk-removed 11 dpf embryos from a phenofemale's clutch (GRZ-$gdf6Y^{del9}$; XX, XY*, XY, YY*; n=4 each). *Gdf6X/Y* probes contain three consecutive RNA pieces covering the *gdf6X* coding sequence in the respective direction. Antisense probes hybridize with *gdf6Y* and $gdf6Y^{del9}$-mRNA due to the high coding sequence identity with *gdf6X* (97.5 and 96.9 %, respectively). **f, h** Scale bars: 200 µm. **a, c–e, g** Source data are provided as a Source Data file.

SUPPLEMENTARY FIG. 7

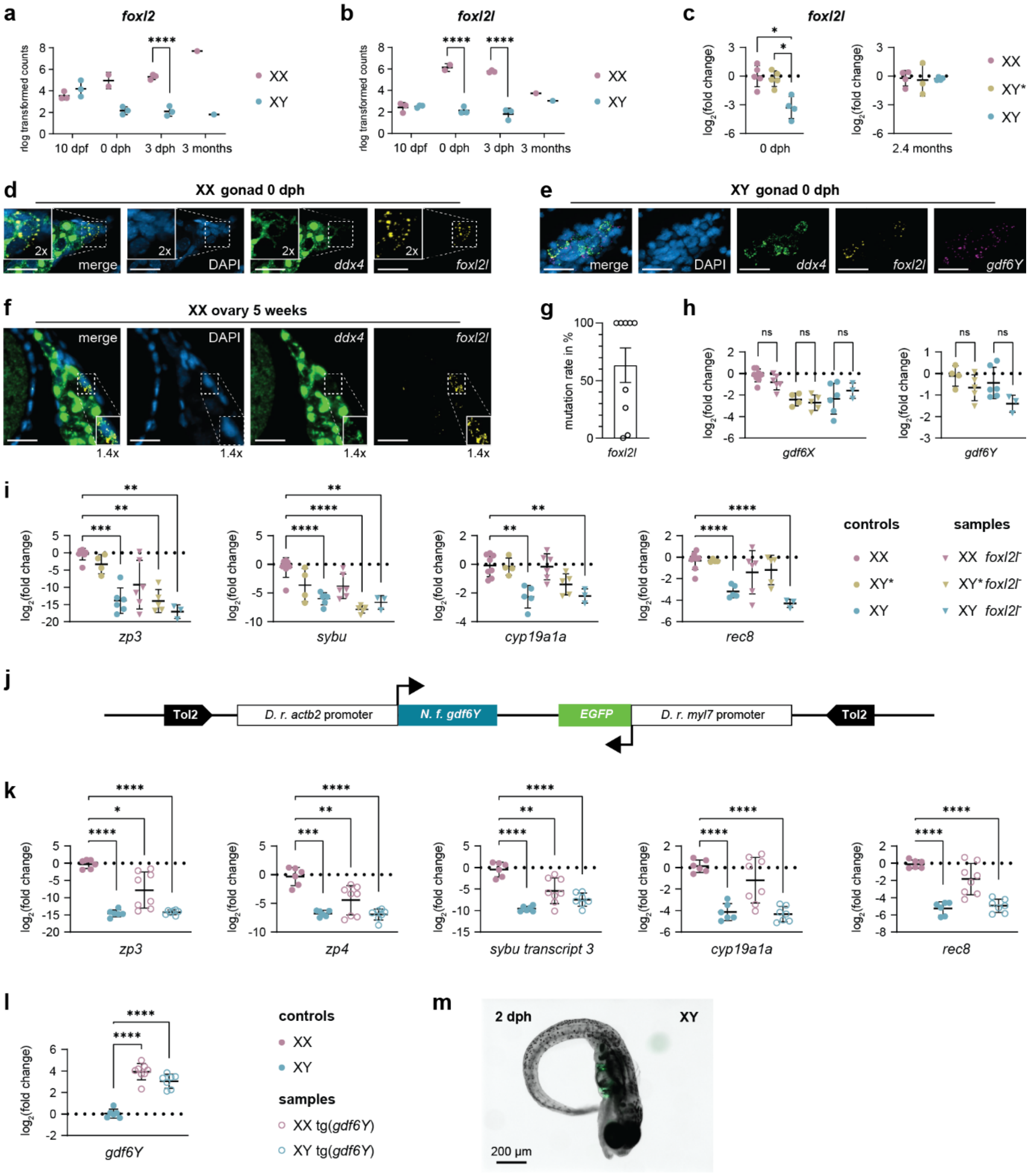

Supplementary Fig. 7 | Expression of *foxl2l* in *N. furzeri* and oogenesis-associated genes in mosaic *foxl2l*-mutants and *gdf6Y*-transgenic animals. RNA-Seq data[1] of (**a**) *foxl2* and (**b**) *foxl2l* from the whole embryo at 10 dpf, the trunk at 0 or 3 dph, and the gonad at 3 months of age in XX and XY animals. Rlog-transformed counts with DESeq2 FDR (Supplementary Data 1). **c** RT-qPCR of *foxl2l* in trunks at 0 dph (XX, XY*, n=5) and adult gonads (2.4 months; XX, n=4; XY*, n=3) of females (XX), phenofemales (XY*), and males (XY; n=4). Welch's ANOVA and Dunnett's T3 multiple comparisons test. **d, e** XX (n=6) and XY (n=1 of 6) gonadal expression at 0 dph of *foxl2l* (yellow), the GC marker *ddx4* (green), and (**e**) *gdf6Y* (magenta). **f** Ovarian expression at 5 weeks of age of *foxl2* (yellow) and the GC marker *ddx4* (green). Nuclei are stained with 4′,6-Diamidin-2-phenylindol (DAPI, blue). **d, e, f** Scale bar: 20 µm. **g** Result of sequence analyses with the Synthego ICE tool (v3.0) of 9 *foxl2l*-mosaic animals created with CRISPR/Cas9. **h** Expression of *gdf6X* and *gdf6Y* and (**i**) female marker genes in trunks of females (XX, n=8), phenofemales (XY*, n=4), and males (XY; *rec8*, *cyp19a1a*, n=5; others, n=6) and their mosaic *foxl2l*$^{-}$ counterparts (XX, n=6; XY*, n=5; XY, n=3) at 0 dph. **j** Schematic of Tol2-transgene to drive ubiquitous *gdf6Y*-expression with a *D. rerio* (*D. r.*) *actb2* promoter in *N. furzeri* (*N. f.*). Heart-specific *EGFP expression* by a *D. r. myl7* promoter served as a selection marker. **k** Expression of female marker genes or (**l**) *gdf6Y* in trunks of females (n=6), males (n=6), and their mosaic *gdf6Y*-transgenic (tg(*gdf6Y*); XX, n=8; XY, n=7) counterparts at 0 dph. **h, i, k, l** Welch's ANOVA and Dunnett's T3 multiple comparisons tests (**h**) between control and CRISPant per sex, (**i, k**) with XX animals, or (**l**) with XY animals. **a–c, i, k, l** (*) P<0.05, (**) P<0.01, (***) P<0.001, (****) P<0.0001. **m** Curled *gdf6Y*-transgenic XY-larva (n=5) with heart-specific *EGFP* expression (green). **c, g–i, k, l** Source data are provided as a Source Data file.

SUPPLEMENTARY FIG. 8

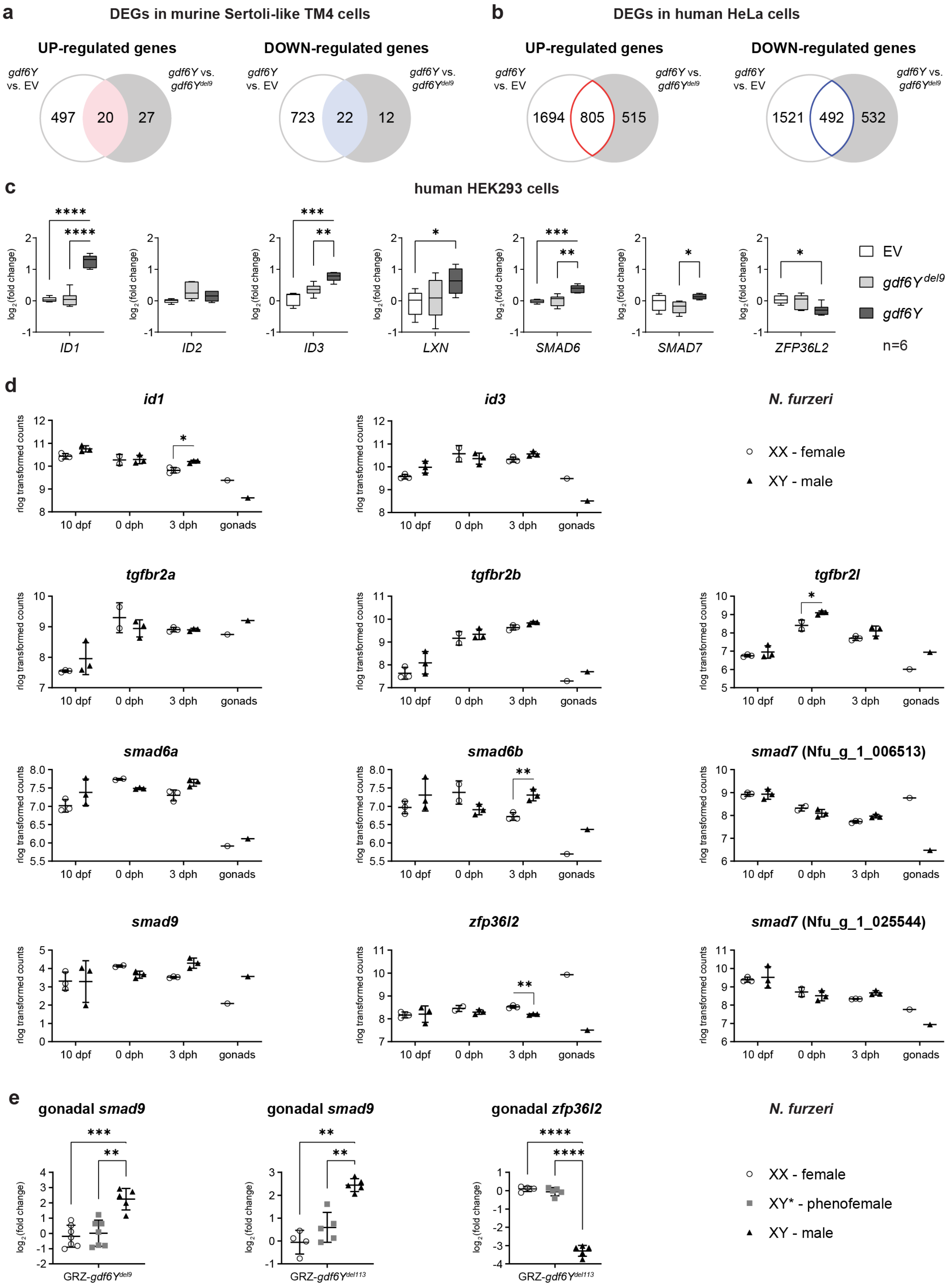

Supplementary Fig. 8 | Gdf6Y-responsive genes in different cell lines and *N. furzeri*. a Overlap of the up- or downregulated DEGs between murine Sertoli-like TM4 cells transfected with either a *gdf6Y* or a *gdf6Y* mutant variant (*gdf6Y^{del9}*) expression plasmid and TM4 cells transfected with either a *gdf6Y* expression plasmid or an empty vector (EV; Supplementary Data 2). **b** Overlap of the up- or downregulated DEGs between human HeLa cells transfected with either a *gdf6Y* or a *gdf6Y* mutant variant (*gdf6Y^{del9}*) expression plasmid and TM4 cells transfected with either a *gdf6Y* expression plasmid or an empty vector (EV; Supplementary Data 3 B-E). **c** RT-qPCR analysis of the genes commonly up- or downregulated in TM4 and HeLa cells in human HEK293 cells transfected with an expression plasmid for *gdf6Y* or a *gdf6Y* mutant variant (*gdf6Y^{del9}*) or an empty vector (EV). Box plots with minimum, 25th percentile, median, 75th percentile, and maximum. Welch's ANOVA and Dunnett's T3 multiple comparisons test with the *gdf6Y* transfected cells. **d** The expression of *id1*, *id3*, *tgfbr2a*, *tgfbr2b*, *tgfbr2l*, *smad6a*, *smad6b*, *smad7* (annotated as two genes), *smad9*, and *zfp36l2* derived from RNA-Seq data[1] in whole embryos at 10 dpf, embryo trunks at 0 or 3 dph and gonads at 3 months of age in female and male *N. furzeri*. Rlog-transformed counts with DESeq2 FDR (Supplementary Data 1). **e** RT-qPCR analysis of the expression of *smad9* and *zfp36l2* in *N. furzeri* gonads of 3 months old females, males, and phenofemales carrying the mutation *gdf6Y^{del9}* (XX, n=7; XY, n=5; XY*, n=7) or *gdf6Y^{del113}* (XX, n=4; XY, n=5; XY*, n=5). Welch's ANOVA and Dunnett's T3 multiple comparisons test. **c–e** (*) $P<0.05$, (**) $P<0.01$, (***) $P<0.001$, (****) $P<0.0001$. **c, e** Source data are provided as a Source Data file.

SUPPLEMENTARY FIG. 9

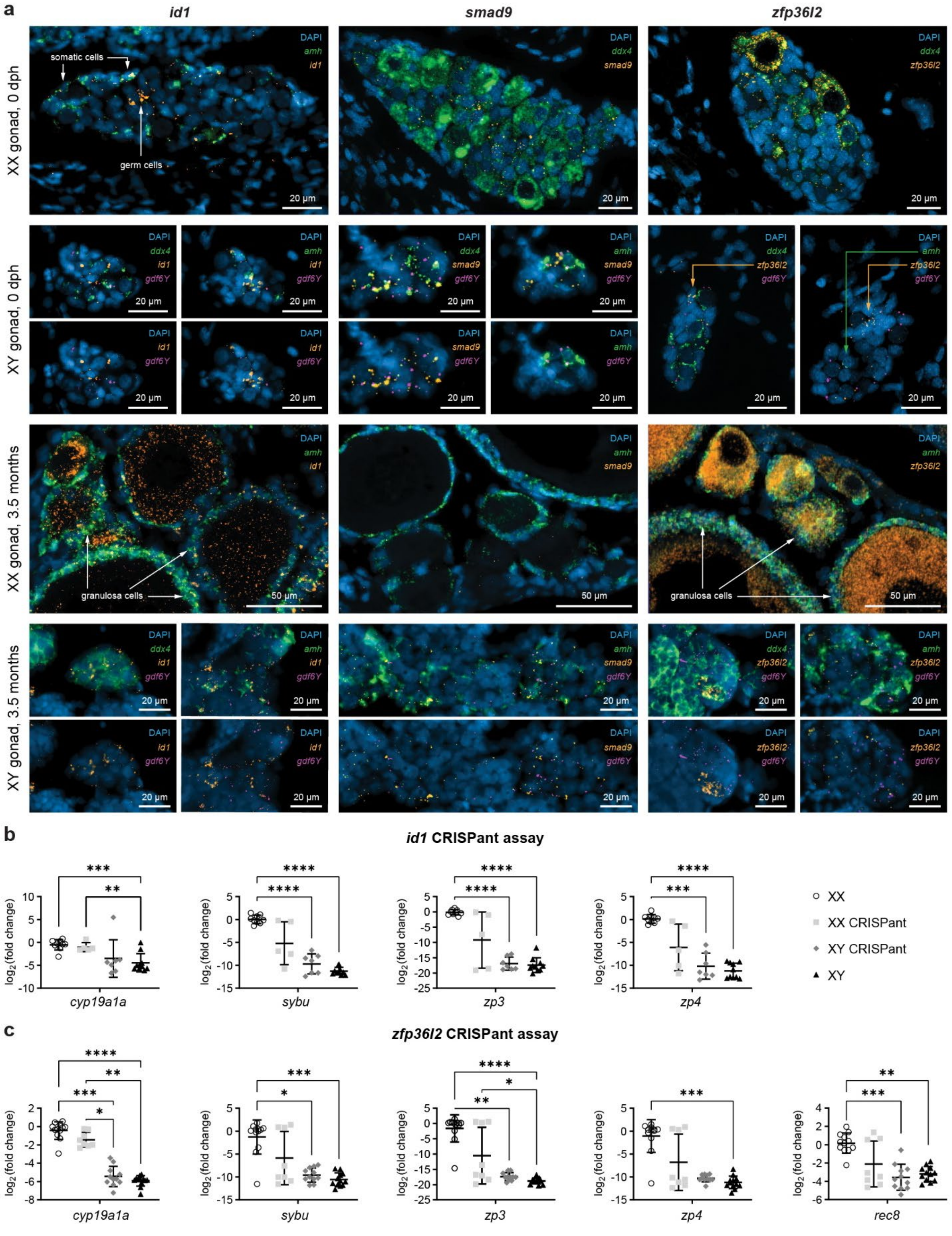

Supplementary Fig. 9 | Gonadal localization and CRISPant assay of Gdf6Y-responsive genes. a Representative RNAscope *in situ* hybridization pictures of XX and XY gonads at the indicated ages (*id1* in XX gonads at 0 dph, n=2; others, n=3) with probes against one out of three Gdf6Y-responsive genes (*id1*, *smad9*, or *zfp36l2*; orange), either the GC marker *ddx4* or the somatic supporting cell marker *amh* (each in green), and *gdf6Y* (magenta) in males. Gamma 0.45 for *zfp36l2* (orange) in XX gonads at 3.5 months. **b** RT-qPCR analyses of female marker genes in trunks of uninjected (n=6 each) and indel-negative (XX, n=3; XY, n=4) females and males and *id1*$^{-}$ CRISPants of both sexes (XX, n=5; XY, n=7) at 0 dph. Welch's ANOVA and Dunnett's T3 multiple comparisons test. **b** RT-qPCR analyses of female marker genes in trunks of uninjected (n=9 each) and indel-negative (n=3 each) females and males and *zfp36l2*$^{-}$ CRISPants of both sexes (XX, n=8; XY, n=11) at 0 dph. Kruskal-Wallis test and Dunn's multiple comparisons test. **b, c** (****) P<0.0001. (**) P<0.01, (***) P<0.001, (****) P<0.0001. Source data are provided as a Source Data file.

SUPPLEMENTARY TABLES

Supplementary Table 1. Mutations of *gdf6Y* and *gdf6X* used in this work.

<table>
<tr><th rowspan="2">Name</th><th colspan="2">Sequence at Cas9 cleavage site (|) with</th><th rowspan="2">(**) Fig.</th></tr>
<tr><td>sgRNA X/Y 1</td><td>sgRNA X/Y 2</td></tr>
<tr><td>gdf6Y</td><td>AAAGG|TCGGGG</td><td>GGTTCTGGACG| TATGGGACGCG</td><td>wild-type</td></tr>
<tr><td>gdf6Y$^{del4, ins3}$</td><td>AAAGG|----GG</td><td>GGTTCTGGACG| CGTTATGGGACGCG</td><td rowspan="3">1f, g; Suppl.: 2a; 5d; 7c (2.4 m)</td></tr>
<tr><td>gdf6Y$^{del4, ins13}$</td><td>AAAGG|----GG</td><td>GGTTCTGGACG| CGTTAAGTCTGATTATGGGACGCG</td></tr>
<tr><td>gdf6Y^{del4}</td><td>AAAGG|TCGGGG</td><td>GGATATGG---| -ATGGGACGCG</td></tr>
<tr><td>gdf6Y$^{del6, del8}$</td><td>AA---|---GGG</td><td>GGT--------| TATGGGACGCG</td><td>1i; Suppl.: 3a, c</td></tr>
<tr><td>gdf6Y^{del9}</td><td>AAAGG|TCGGGG</td><td>GGTTCTGGACG| ---------CG</td><td>1h, i; 2i; 4e; 6d; Suppl.: 2b, d, f, h; 3d, g; 6h; 7h, i; 8c, e</td></tr>
<tr><td>gdf6Y^{del113}</td><td>AAAGG|------</td><td>-----------| ----GGACGCG</td><td>Suppl.: 2c, e, g; 3b; 4b; 5c; 6a; 7c (0 dph); 8e</td></tr>
<tr><td>(*) gdf6Y^{ins25}</td><td>AAAGG|TCGGGG</td><td>GGTTCTGGACG|CGTTTAAAGCACATCGGCTGGGAGGTTCTGGACGCG</td><td>2i</td></tr>
<tr><td>gdf6X</td><td>AAAAG|TCGGGG</td><td>GGTTCTGGATG| TATGGGACGCG</td><td>wild-type</td></tr>
<tr><td>gdf6X^{del113}</td><td>AAA--|------</td><td>-----------| --TGGGACGCG</td><td>2c-h; Suppl.: 3h-j</td></tr>
</table>

(*) Artificial mutation used in *in vitro* analysis. (**) Suppl. – Supplementary.

Supplementary Table 2. Oligonucleotides used for the synthesis of sgRNA *in vitro* transcription templates.

Name	Sequence (sgRNA target bold)	Application
sgRNA_Gdf6_2_fw	TAGG**AAGGGACTGTGCAAAGGTCG**	Cloning of sgRNA Y1
sgRNA_Gdf6_2_rv	AAAC**CGACCTTTGCACAGTCCCTT**	
sgRNA_Gdf6_4_fw	TAGG**CGGCTGGGAGGTTCTGGACGTAT**	Cloning of sgRNA Y2
sgRNA_Gdf6_4_rv	AAAC**ATACGTCCAGAACCTCCCAGCCG**	
T7sgRgdf6X1F	GAAATTAATACGACTCACTATAGG**AAGGGAATGCGCAAAAGTCG** GTTTTAGAGCTAGAAATAG	Amplification of sgRNA X1
T7sgRgdf6X2F	GAAATTAATACGACTCACTATAGG**CGGCTGGGAGGTTCTGGATGTAT** GTTTTAGAGCTAGAAATAG	Amplification of sgRNA X2
T7sgRfoxl2l2F	GAAATTAATACGACTCACTATAGG**GAAGCCCCCCTATTCCTACG** GTTTTAGAGCTAGAAATAG	Template for *foxl2l*-sgRNA1
T7sgRfoxl2l3F	GAAATTAATACGACTCACTATAGG**AAGGTGCCCCGAGAGCGCGG** GTTTTAGAGCTAGAAATAG	Template for *foxl2l*-sgRNA2
BAC_target1-sgRF	GAAATTAATACGACTCACTATAGG**GATAAACGTAGACTAAAACG** GTTTTAGAGCTAGAAATAG	Template for BAC-sgRNA
Nf_clyblsgR2rvF	GAAATTAATACGACTCACTATAGG**GATGTGTGTACAGTCCACAG** GTTTTAGAGCTAGAAATAG	Template for *clybl*-sgRNA
Nf_id1sgR1F	GAAATTAATACGACTCACTATAGG**TCTTGCACTTGGAGATGGCG** GTTTTAGAGCTAGAAATAG	Template for *id1*-sgRNA1
Nf_id1sgR2F	GAAATTAATACGACTCACTATAGG**CACAAACAAGAAGGCCAGCA** GTTTTAGAGCTAGAAATAG	Template for *id1*-sgRNA2
Nf_zfp36l2sgR1F	GAAATTAATACGACTCACTATAGG**GTTCCCCTTGTTCATGATGG** GTTTTAGAGCTAGAAATAG	Template for *zfp36l2*-sgRNA1
Nf_zfp36l2sgR2F	GAAATTAATACGACTCACTATAGG**GGTTTTGTCCCTATGGTCCC** GTTTTAGAGCTAGAAATAG	Template for *zfp36l2*-sgRNA2
sgRNAR	AAAAGCACCGACTCGGTGCCACTTTTTCAAGTTGATAACGGACTAGC CTTATTTTAACTTGCTATTTCTAGCTCTAAAAC	Template for sgRNAs
T7F	GAAATTAATACGACTCAC	Amplification sgRNAs
pDRrvs	AAAAGCACCGACTCGGTGC	

Supplementary Table 3. Numbers of injected zygotes for experiments conducted exclusively in F0.

Experiment	Injected zygotes	Surviving embryos until dissection	Fig./Supplementary (Suppl.) Fig.
foxl2l-inactivation	414	32	4e; Suppl.: 7g-i
tg(*gdf6Y*)-expression RT-qPCR	324	23 (17 transgene-positive)	Suppl.: 7k, l
tg(*gdf6Y*)-expression hatch	461	23 (5 transgene-positive)	Suppl.: 7m
gdf6Y-BAC transgenesis	324	20	5b-f
id1-inactivation	1291	19	6e; Suppl.: 9b
zfp36l2-inactivation	593	32	6f; Suppl.: 9c

Supplementary Table 4. Oligonucleotides used for sexing and genotyping.

Name	Sequence	Annealing T [°C]	Usage
Nf_Gdf6m_fw4	GAGAAAGAGGAGCTGGTCGGG	64	*gdf6Y*-specific PCR
Nf_Gdf6Y_rv4	GCGCTGCTGATGATGATGCC		
sgr5-delF1	TGCAAGACAGTCTTCCATTCAGGG	63	sexing PCR[2]
sgr5-delR2	CCTTGAGGACGGCTGCTTCC		
bckdhb_intron2F	TTCCTGACGGCGATCTGAGT	60	sexing-PCR outside BAC-cloned Y-chromosomal sequence
bckdhb_intron2R	CATGACACATCTGCGTGACC		
gdf6-gtF2	GGCCTCTGTGGGAGAATGTG	60	pan-*gdf6*-PCR
gdf6-gtR1	ACCGACCTCCGCGACTTG		
foxl2lt2F	GAGGTATGGATGCGGATAAGAA	60	*foxl2l*-PCR
foxl2lt3R	CTGATGCAGGTAGTAGTCGGTG		
clybl_2F1	CCACCATCTTTGACCTGATTTT	58	*clybl*-PCR
clybl_2R	GTTGTGTATATGCGACACAGCA		
NEB-id1F	TTTAAACTTAAGCTTCCGCCATGAAGGTTGTTGGATCTACCTG	60	*id1*-PCR
NEB-id1R	CTGGATATCTGCAGAATTCCTTAGCGGCACATTATCCTGTCGT<br>CCGAGCATCCGTTCTCCACTGAGATACTGGCG		
zfp36l2genoF2	GCAGAAACTCCACCAGCAAC	60	*zfp36l2*-PCR
zfp36l2genoR2	GTTGGCGTTCGCAGCAGG		
gdf6YinsituF1	CGGAGGGTAACCTGCTGC	55	Tol2-transgene-specific PCR
M13F	GTAAAACGACGGCCAG		
BAC-CmR_F2	TTAGGGAAATAGGCCAGGTTTT	58	BAC-PCR
BAC-CmR_R1	TTGTTACACCGTTTTCCATGAG		
scaffold795_4r	AAATCCTCTCCTGGCCTACC	59	MZM0403-sexing
scaffold795_3f	GGCTGGCGAGAGTCTTTCTA		

Supplementary Table 5. RNAscope probes purchased from Advanced Cell Diagnostics.

Name	Targeted transcript (NCBI Reference Sequence)	Catalogue-No.
RNAscope® Probe - Nf-ddx4	XM_015957842.1 (*ddx4*)	563361
RNAscope® Probe - Nf-amh	XM_015977279.1 (*amh*)	830891
RNAscope® Probe - Nf-amh-C2	XM_015977279.1 (*amh*)	830891-C2
RNAscope® Probe - Nf-LOC107386104-C2	XM_015960292.1 (*dmrt1*)	830901-C2
RNAscope® Probe - Nf-LOC107389222-C2	XM_015965254.1 (*wt1a*)	830871-C2
RNAscope® Probe - Nf-LOC107390636-C2	XM_015967458.1 (*foxl2l*)	831201-C2
RNAscope® Probe - Nf-gdf6y-C3	*gdf6Y*-transcript[1]	563381-C3
RNAscope® Probe - Nf-zfp36l2-C2	XM_015944101.2 (*zfp36l2*)	1274961-C2
RNAscope® Probe - Nf-smad9-C2	XM_015968326.2 (*smad9*)	1274971-C2
RNAscope® Probe - Nf-id1-C2	XM_015960037.2 (*id1*)	1274981-C2

Supplementary Table 6. Oligonucleotides used for RT-qPCR.

Name	Sequence	Usage (Fig./Supplementary [Suppl.] Fig.)
qRPL13a_ex4/5_fw	ACTGTCAGAGGCATGCTTCC	Quantification of *rpl13a* as universal *N. furzeri* reference gene
qRPL13a_ex6_rv	GGCGCACAATTTTCAGAGCA	
eef1a1l1F	GAAGGAAGCCGCCGAGATG	Quantification of *eef1a1l1* as 2nd *N. furzeri* reference gene (4e *tacc3*; 6d *id1*; Suppl.: 6g; 7i (*cyp19a1a*, *rec8*), k, l)
eef1a1l1R	CAATGATGGTCACGTAGTACTTG	
cyp19a1aqF1	TTGGGATGCATGAGAGAGGC	Quantification of *cyp19a1a* in adult *N. furzeri* gonads
cyp19a1aqR1	GAGTCTGTGTGGAGGAGATGC	
cyp19a1aF2	GCAGATCTCATATTTGCACAAAACC	Quantification of *cyp19a1a* in *N. furzeri* trunks at 0 dph
cyp19a1aR2	CGTTCTGAAGCTCTTCTCCTAC	
dmrt1ex3qF1	CTGTGGAAGGACGTTCCAGC	Quantification of *dmrt1* in *N. furzeri*
dmrt1ex4qR1	GTCTCCAGCAGCAGGTCAG	
foxl2-qRTF	GGTCCAGGAGAAAGTCACGG	Quantification of *foxl2* in *N. furzeri*
foxl2-qRTR	GCTGTTCTGCCATCCCTTCT	
foxl3-qRTF1	GACCGGAAAAGCAACTTCTG	Quantification of *foxl2l* in *N. furzeri* trunks at 0 dph
foxl3-qRTR1	CGGTCATGTAAGGCACACTG	
foxl3-qRTF2	GATCCCGCATTTGAAGACAT	Quantification of *foxl2l* in adult *N. furzeri* gonads
foxl3-qRTR2	GGAGCCGCTCACATACTGAT	
Nf_Gdf6a_fw1	TTCCGCTCCTCTAAATCTGC	Quantification of *gdf6X* & *gdf6Y* in *N. furzeri* (1f; Suppl.: 2e, f)
Nf_Gdf6a_rv1	TTCCTGAAAATCCGCAGTTC	
gdf6ex1qF1	CCATAACGAGTTTTGTGGACAGAGG	Quantification of *gdf6X* & *gdf6Y* in *N. furzeri* (Suppl.: 4a; 6g)
gdf6ex2qR1	CTGAAAATCCGCAGTTCCGC	
gdf6ex1qF1	CCATAACGAGTTTTGTGGACAGAGG	Quantification of *gdf6X* in *N. furzeri* (Suppl.: 4a; 7h)
Nf_Gdf6_rv4	GCGCTGCTGCTGATGATAGT	
gdf6ex1qF1	CCATAACGAGTTTTGTGGACAGAGG	Quantification of *gdf6Y* in *N. furzeri* (Suppl.: 4a; 6g; 7h)
Nf_Gdf6Y_rv4	GCGCTGCTGATGATGATGCC	
gdf6Y_3UTR_F1	ACTGAACGATTAATCCACCAGAC	Quantification of *gdf6Y* in *N. furzeri* (1f)
gdf6/Y_3UTR_R11	ATAATAATGAACAGCAGTGCATTC	
Nf_id1-qRTf2	GCCAGTATCTCAGTGGAGAACG	Quantification of *id1* in *N. furzeri*
Nf_id1-qRTr2	GCTGGTTGAGACCATCTCGG	
KDM6B-F3	CAGACCACGCTACGGAAAAAG	Quantification of Nfu_g_1_015428 in *N. furzeri*
KDM6B-R23	CCTCCGCATCTGCCTCC	
LOC100572914-F6	AAGGACCATAGTGGCTGTCG	Quantification of Nfu_g_1_014802 in *N. furzeri*
LOC100572914-R5	CGAGGTCTTCACAGCACGTTT	
PCTPfw3	GGTAAACTGGGCAGCAAAGAG	Quantification of *pctp* in *N. furzeri*
PCTPrv3	GGTGGACACTTTCCCATCAG	
rec8F2	TTACCACCAGAGGCAAACCG	Quantification of *rec8* in *N. furzeri*
rec8R2	GATGTCTCCAAACGGCTCATG	
rnf19a_fwd1	TGGTGCCCATCTCTCTGTGT	Quantification of *rnf19a* in *N. furzeri*
rnf19a_rev1	TCTGCTACTGATGTCATGTCGG	
smad9qRTF1	GTAAGATCCCCAGTGGCTGC	Quantification of *smad9* in *N. furzeri*
smad9qRTR1	TCCTGACGGTGGTATTCAGC	
Sybu_fw1	CAACCCAGAGCAGTACCTGA	Quantification of all *sybu* transcript variants in *N. furzeri*
Sybu_rv1	TCTTCCCTCATTCTGCCAAG	
Sybu_fw7	AAGCTGCTGTGTGAAGCAGA	Quantification of *sybu* transcript variant 1 & 2 in *N. furzeri*
Sybu_rv2	CCTCTTCACTTTGGCTTTGC	
Sybu_fw9	GGAAGATCACTGAGTTTTGACC	Quantification of *sybu* transcript variant 3 in *N. furzeri*
Sybu_rv3	CAGGTACTGCTCTGGGTTGG	
TACC3_F2	ACCAACAGCTGAGCCCAAC	Quantification of *tacc3* in *N. furzeri*
TACC3_R2	TAGCTGCTGATCGGGCTG	
FIZ1-F3	CAGGACGATCACTCAGAACAAG	Quantification of *zfp2* in *N. furzeri*
FIZ1-R13	GCTCACTCTGCTCCTCTTC	
zfp36l2qRTF2	CGTCCAAACCGTGCTTCTTG	Quantification of *zfp36l2* in *N. furzeri*
zfp36l2qRTR2	TCTTTAGCGCACAGCTGGAA	
ZPC-F2	GACTGACCCCCGATCACAG	Quantification of *zp3* in *N. furzeri*
ZPC-R2	CTCATCTCCAGTAACCAGTTTCC	
ZPB_F	GGACCAAGAGGATCCATCAC	Quantification of *zp4* in *N. furzeri*
ZPB_R	CCCTGCAGCCGCCAC	
HsPUM1qRTF	TGCGGGAGATTGCTGGACAT	Quantification of *PUM1* as universal human reference gene[3]
HsPUM1qRTR	GTGTGGCACGCTCCAGTTTC	
HsID1qRTF1	AGAACCGCAAGGTGAGCAAGG	Quantification of human *ID1*
HsID1qRTR1	AGGAACGCATGCCGCCTCG	
HsID2qRTF	CCTGCATCACCAGAGACCCG	Quantification of human *ID2*
HsID2qRTR	CAGAAGGGAATTCAGAAGCCTGC	
HsID3qRTF	CCTGACACCTCCAGAACGCA	Quantification of human *ID3*
HsID3qRTR	GAAGTTGGGGCCCATCCCTG	
HsLXNqRTF	GCCTGGGTTGCCTGTGGTTA	Quantification of human *LXN*
HsLXNqRTF	TGCCAGAGAACTTGCATTTGCC	
HsSMAD6qRTF	CGCGACGAGTACAAGCCACT	Quantification of human *SMAD6*
HsSMAD6qRTR	CGGAGACATGCTGGCGTCTG	
HsSMAD7qRTF	TGCAACCCCCATCACCTTAGC	Quantification of human *SMAD7*
HsSMAD7qRTR	CCCCCTGTTTCAGCGGAGGA	
HsZFP36L2qRTF	CAAGGGCTCCTCCCGACCTC	Quantification of human *ZFP36L2*
HsZFP36L2qRTR	CCAGGGATTTCTCTGTCTTGCAC	

Supplementary Table 7. Oligonucleotides used for WISH probe generation.

Name	Sequence	Usage
Nf_sybu_fw2	AGAGGACCATCAGCACCAAC	*sybu*-probe cloning
Nf_sybu_rv6	CAGGGACTCCAACTTCCT	
gdf6OrF	**ATGGACGCATCTCGAGTCG**	Cloning of the 5'-part of *gdf6X*'s CDS
gdf6XYsoeR	GATTGTCTGTTCCTCTGTCCACAAAACTCG	
Nf_Gdf6a_fw2	TCTCCTCTGCGAAGACAAAAG	Cloning of the central part of *gdf6X*'s CDS
gdf6-gtR2	CGGCTTCTCCTCCTTTTCACC	
gdf6XYsoeF	GGAGGAGGACGGCGCTG	Cloning of the 3'-part of *gdf6X*'s CDS
gdf6StopR	CTACCTGCAGCCGCACTG	
M13F	GTAAAACGACGGCCAG	Amplification of *gdf6X*'s probe
M13R	CAGGAAACAGCTATGAC	

Supplementary Table 8. Oligonucleotides used for bisulfite sequencing.

Name	Sequence of oligonucleotide f	Sequence of oligonucleotide r
ATG COD *gdf6* 1	ATGATTTAAAAGTTGTTGGAATAAATTTTATTT	ATGTTATTAACTAATACACCAAAATTAAAAAAC
ATG COD *gdf6* 2	ATGGTTAATAAATATTTTTAGAGGTTAGTAGAG	ATGACCCTAACAAATCTAATCACACCA
ATG COD *gdf6* 3	ATGGGTGTGATTAGATTTGTTAGGGT	ATGAACATCTCTACAAACAAATTATATTCTAAA
ATG COD *gdf6* 4	ATGTTAATGGATTTTTAGAATATAATTTGTTTG	ATGTTACTCTAAACTCTTCATAAAAACAAAAAC
ATG COD *gdf6* 6	ATGATTAGTTTGAAGTTTTGTATTAGGTTATTA	ATGTCTAATTTATAAATCCACCAAAACC
ATG transposon 1	ATGTTGTAATAGATTGAAATGTAATGTAATTTA	ATGTATATCTCTCACCTAACCAAAAAAC
ATG transposon 2	ATGAGGTAGTGGTTAATTGATATTTT	ATGTTTATCTAACATATCTAAACCTAATAATAA
ATG transposon 3	ATGGTTTTGGTTTTTGGTTTTTGTTAGT	ATGAAATAACCTATAAAAATTTTATACATTT
ATG COD *sybu* 1	ATGAATGTTTGAAGTTGTTGATTTTAGT	ATGAATCTATTTCCAACTCTCCTCC
GCA COD *sybu* 2	GCAGGAGAGTTGGAAATAGATTGA	GCAAATACTAAACAAAAACCAAATTTAAATT
GCA COD *sybu* 3	GCATGATGTTATTTATAAGATTTGGTTTATT	GCACTTTCATATTATTCTACATTTAAACAACTA
GCA NON COD *gdf6* 1	GCAAGTTTATGAAGGGAATTTGGATTG	GCATCAAAAACTATTAAAACAAACTTTACTCTA
GCA NON COD *gdf6* 2	GCAGAGTTTTTTTGATTAGTAAGGTTGTT	GCAAAACTCATTTATAATCCAACCATTTAAAC
GCA NON COD *gdf6* 4	GCATTTTTATAAGAGTAGAGATTGTTGGGTAGAG	GCACTCAATAAATTCTCAAAATATAATCTATTT
GCA NON COD *sybu* 1	GCAGAAGGTTGGGATATTTTGTG	GCAAAATATCTAAAATTATTAATTTCAATC
GCA NON COD *sybu* 2	GCAATTTTATAAATGATATTAGATATTGAATAA	GCAAAAAAAAACTAAAAACAAACTAAC
GCA NON COD *sybu* 3	GCATATTTTATATTTGGATAGTTGGGGG	GCAATTCTATATCAACACAAACAAAAAAAC
GCA NON COD *sybu* 4	GCAGATAGTGTTTTAGTATTATGGAGAG	GCAATCATTTATAAAATTTAATCCATCC
CAT COD *gdf6* 1	CATATTTAAAAGTTGTTGGAATAAATTTTATTT	CATTTATTAACTAATACACCAAAATTAAAAAAC
CAT COD *gdf6* 2	CATGTTAATAAATATTTTTAGAGGTTAGTAGAG	CATACCCTAACAAATCTAATCACACCA
CAT COD *gdf6* 3	CATGGTGTGATTAGATTTGTTAGGGT	CATAACATCTCTACAAACAAATTATATTCTAAA
CAT COD *gdf6* 4	CATTTAATGGATTTTTAGAATATAATTTGTTTG	CATTTACTCTAAACTCTTCATAAAAACAAAAAC
CAT COD *gdf6* 6	CATATTAGTTTGAAGTTTTGTATTAGGTTATTA	CATTCTAATTTATAAATCCACCAAAACC
CAT transposon 1	CATTTGTAATAGATTGAAATGTAATGTAATTTA	CATTATATCTCTCACCTAACCAAAAAAC
CAT transposon 2	CATAGGTAGTGGTTAATTGATATTTT	CATTTTATCTAACATATCTAAACCTAATAATAA
CAT transposon 3	CATGTTTTGGTTTTTGGTTTTTGTTAGT	CATAAATAACCTATAAAAATTTTATACATTT
CAT COD *sybu* 1	CATAATGTTTGAAGTTGTTGATTTTAGT	CATAATCTATTTCCAACTCTCCTCC
TGC COD *sybu* 2	TGCGGAGAGTTGGAAATAGATTGA	TGCAATACTAAACAAAAACCAAATTTAAATT
TGC COD *sybu* 3	TGCTGATGTTATTTATAAGATTTGGTTTATT	TGCCTTTCATATTATTCTACATTTAAACAACTA
TGC NON COD *gdf6* 1	TGCAGTTTATGAAGGGAATTTGGATTG	TGCTCAAAAACTATTAAAACAAACTTTACTCTA
TGC NON COD *gdf6* 2	TGCGAGTTTTTTTGATTAGTAAGGTTGTT	TGCAAACTCATTTATAATCCAACCATTTAAAC
TGC NON COD *gdf6* 4	TGCTTTTTATAAGAGTAGAGATTGTTGGGTAGAG	TGCCTCAATAAATTCTCAAAATATAATCTATTT
TGC NON COD *sybu* 1	TGCGAAGGTTGGGATATTTTGTG	TGCAAATATCTAAAATTATTAATTTCAATC
TGC NON COD *sybu* 2	TGCATTTTATAAATGATATTAGATATTGAATAA	TGCAAAAAAAACTAAAAACAAACTAAC
TGC NON COD *sybu* 3	TGCTATTTTATATTTGGATAGTTGGGGG	TGCATTCTATATCAACACAAACAAAAAAAC
TGC NON COD *sybu* 4	TGCGATAGTGTTTTAGTATTATGGAGAG	TGCATCATTTATAAAATTTAATCCATCC

Supplementary Table 9. Composition of the 8 amplicon pools destined for Illumina tagging.

#	Pool 1		Pool 2		Pool 3		Pool 4	
	**n**	**Oligonucleotides f/r**	**n**	**Oligonucleotides f/r**	**n**	**Oligonucleotides f/r**	**n**	**Oligonucleotides f/r**
1	F1	ATG COD *gdf6* 1	F3	ATG COD *gdf6* 1	F5	ATG COD *gdf6* 1	M1	TGC NON COD *sybu* 4
2	F1	ATG COD *gdf6* 2	F3	ATG COD *gdf6* 2	F5	ATG COD *gdf6* 2	M2	ATG COD *gdf6* 1
3	F1	ATG COD *gdf6* 3	F3	ATG COD *gdf6* 3	F5	ATG COD *gdf6* 3	M2	ATG COD *gdf6* 2
4	F1	ATG COD *gdf6* 4	F3	ATG COD *gdf6* 4	F5	ATG COD *gdf6* 4	M2	ATG COD *gdf6* 3
5	F1	ATG COD *gdf6* 6	F3	ATG COD *gdf6* 6	F5	ATG COD *gdf6* 6	M2	ATG COD *gdf6* 4
6	F1	ATG transposon 1	F3	ATG transposon 1	F5	ATG transposon 1	M2	ATG COD *gdf6* 6
7	F1	ATG transposon 3	F3	ATG transposon 3	F5	ATG transposon 3	M2	ATG transposon 1
8	F1	ATG COD *sybu* 1	F3	ATG COD *sybu* 1	F5	ATG COD *sybu* 1	M2	ATG transposon 2
9	F1	GCA COD *sybu* 2	F3	GCA COD *sybu* 2	F5	GCA COD *sybu* 2	M2	ATG transposon 3
10	F1	GCA COD *sybu* 3	F3	GCA COD *sybu* 3	F5	GCA COD *sybu* 3	M2	ATG COD *sybu* 1
11	F1	GCA NON COD *gdf6* 1	F3	GCA NON COD *gdf6* 1	F5	GCA NON COD *gdf6* 1	M2	GCA COD *sybu* 2
12	F1	GCA NON COD *gdf6* 2	F3	GCA NON COD *gdf6* 2	F5	GCA NON COD *gdf6* 2	M2	GCA COD *sybu* 3
13	F1	GCA NON COD *gdf6* 4	F3	GCA NON COD *gdf6* 4	F5	GCA NON COD *gdf6* 4	M2	GCA NON COD *gdf6* 1
14	F1	GCA NON COD *sybu* 1	F3	GCA NON COD *sybu* 1	F5	GCA NON COD *sybu* 1	M2	GCA NON COD *gdf6* 2
15	F1	GCA NON COD *sybu* 2	F3	GCA NON COD *sybu* 2	F5	GCA NON COD *sybu* 2	M2	GCA NON COD *gdf6* 4
16	F1	GCA NON COD *sybu* 3	F3	GCA NON COD *sybu* 3	F5	GCA NON COD *sybu* 3	M2	GCA NON COD *sybu* 1
17	F1	GCA NON COD *sybu* 4	F3	GCA NON COD *sybu* 4	F5	GCA NON COD *sybu* 4	M2	GCA NON COD *sybu* 2
18	F2	CAT COD *gdf6* 1	F4	CAT COD *gdf6* 1	M1	CAT COD *gdf6* 1	M2	GCA NON COD *sybu* 3
19	F2	CAT COD *gdf6* 2	F4	CAT COD *gdf6* 2	M1	CAT COD *gdf6* 2	M2	GCA NON COD *sybu* 4
20	F2	CAT COD *gdf6* 3	F4	CAT COD *gdf6* 3	M1	CAT COD *gdf6* 3	M3	CAT COD *gdf6* 1
21	F2	CAT COD *gdf6* 4	F4	CAT COD *gdf6* 4	M1	CAT COD *gdf6* 4	M3	CAT COD *gdf6* 2
22	F2	CAT COD *gdf6* 6	F4	CAT COD *gdf6* 6	M1	CAT COD *gdf6* 6	M3	CAT COD *gdf6* 3
23	F2	CAT transposon 1	F4	CAT transposon 1	M1	CAT transposon 1	M3	CAT COD *gdf6* 4
24	F2	CAT transposon 3	F4	CAT transposon 3	M1	CAT transposon 2	M3	CAT COD *gdf6* 6
25	F2	CAT COD *sybu* 1	F4	CAT COD *sybu* 1	M1	CAT transposon 3	M3	CAT transposon 1
26	F2	TGC COD *sybu* 2	F4	TGC COD *sybu* 2	M1	CAT COD *sybu* 1	M3	CAT transposon 2
27	F2	TGC COD *sybu* 3	F4	TGC COD *sybu* 3	M1	TGC COD *sybu* 2	M3	CAT transposon 3
28	F2	TGC NON COD *gdf6* 1	F4	TGC NON COD *gdf6* 1	M1	TGC COD *sybu* 3	M3	CAT COD *sybu* 1
29	F2	TGC NON COD *gdf6* 2	F4	TGC NON COD *gdf6* 2	M1	TGC NON COD *gdf6* 1	M3	TGC COD *sybu* 2
30	F2	TGC NON COD *gdf6* 4	F4	TGC NON COD *gdf6* 4	M1	TGC NON COD *gdf6* 2	M3	TGC COD *sybu* 3
31	F2	TGC NON COD *sybu* 1	F4	TGC NON COD *sybu* 1	M1	TGC NON COD *gdf6* 4	M3	TGC NON COD *gdf6* 1
32	F2	TGC NON COD *sybu* 2	F4	TGC NON COD *sybu* 2	M1	TGC NON COD *sybu* 1	M3	TGC NON COD *gdf6* 2
33	F2	TGC NON COD *sybu* 3	F4	TGC NON COD *sybu* 3	M1	TGC NON COD *sybu* 2	M3	TGC NON COD *gdf6* 4
34	F2	TGC NON COD *sybu* 4	F4	TGC NON COD *sybu* 4	M1	TGC NON COD *sybu* 3	M3	TGC NON COD sybu 1
**#**	**Pool 5**		**Pool 6**		**Pool 7**		**Pool 8**	
	**n**	**Oligonucleotides f/r**	**n**	**Oligonucleotides f/r**	**n**	**Oligonucleotides f/r**	**n**	**Oligonucleotides f/r**
1	M3	TGC NON COD *sybu* 2	M5	TGC NON COD *gdf6* 4	P2	TGC NON COD *gdf6* 1	P4	TGC COD *sybu* 2
2	M3	TGC NON COD *sybu* 3	M5	TGC NON COD *sybu* 1	P2	TGC NON COD *gdf6* 2	P4	TGC COD *sybu* 3
3	M3	TGC NON COD *sybu* 4	M5	TGC NON COD *sybu* 2	P2	TGC NON COD *gdf6* 4	P4	TGC NON COD *gdf6* 1
4	M4	ATG COD *gdf6* 1	M5	TGC NON COD *sybu* 3	P2	TGC NON COD *sybu* 1	P4	TGC NON COD *gdf6* 2
5	M4	ATG COD *gdf6* 2	M5	TGC NON COD *sybu* 4	P2	TGC NON COD *sybu* 2	P4	TGC NON COD *gdf6* 4
6	M4	ATG COD *gdf6* 3	P1	ATG COD *gdf6* 1	P2	TGC NON COD *sybu* 3	P4	TGC NON COD *sybu* 1
7	M4	ATG COD *gdf6* 4	P1	ATG COD *gdf6* 2	P2	TGC NON COD *sybu* 4	P4	TGC NON COD *sybu* 2
8	M4	ATG COD *gdf6* 6	P1	ATG COD *gdf6* 3	P3	ATG COD *gdf6* 1	P4	TGC NON COD *sybu* 3
9	M4	ATG transposon 1	P1	ATG COD *gdf6* 4	P3	ATG COD *gdf6* 2	P4	TGC NON COD *sybu* 4
10	M4	ATG transposon 2	P1	ATG COD *gdf6* 6	P3	ATG COD *gdf6* 3	P5	ATG COD *gdf6* 1
11	M4	ATG transposon 3	P1	ATG transposon 1	P3	ATG COD *gdf6* 4	P5	ATG COD *gdf6* 2
12	M4	ATG COD *sybu* 1	P1	ATG transposon 2	P3	ATG COD *gdf6* 6	P5	ATG COD *gdf6* 3
13	M4	GCA COD *sybu* 2	P1	ATG transposon 3	P3	ATG transposon 1	P5	ATG COD *gdf6* 4
14	M4	GCA COD *sybu* 3	P1	ATG COD *sybu* 1	P3	ATG transposon 2	P5	ATG COD *gdf6* 6
15	M4	GCA NON COD *gdf6* 1	P1	GCA COD *sybu* 2	P3	ATG transposon 3	P5	ATG transposon 1
16	M4	GCA NON COD *gdf6* 2	P1	GCA COD *sybu* 3	P3	ATG COD *sybu* 1	P5	ATG transposon 2
17	M4	GCA NON COD *gdf6* 4	P1	GCA NON COD *gdf6* 1	P3	GCA COD *sybu* 2	P5	ATG transposon 3
18	M4	GCA NON COD *sybu* 1	P1	GCA NON COD *gdf6* 2	P3	GCA COD *sybu* 3	P5	ATG COD *sybu* 1
19	M4	GCA NON COD *sybu* 2	P1	GCA NON COD *gdf6* 4	P3	GCA NON COD *gdf6* 1	P5	GCA COD *sybu* 2
20	M4	GCA NON COD *sybu* 3	P1	GCA NON COD *sybu* 1	P3	GCA NON COD *gdf6* 2	P5	GCA COD *sybu* 3
21	M4	GCA NON COD *sybu* 4	P1	GCA NON COD *sybu* 2	P3	GCA NON COD *gdf6* 4	P5	GCA NON COD *gdf6* 1
22	M5	CAT COD *gdf6* 1	P1	GCA NON COD *sybu* 3	P3	GCA NON COD *sybu* 1	P5	GCA NON COD *gdf6* 2
23	M5	CAT COD *gdf6* 2	P1	GCA NON COD *sybu* 4	P3	GCA NON COD *sybu* 2	P5	GCA NON COD *gdf6* 4
24	M5	CAT COD *gdf6* 3	P2	CAT COD *gdf6* 1	P3	GCA NON COD *sybu* 3	P5	GCA NON COD *sybu* 1
25	M5	CAT COD *gdf6* 4	P2	CAT COD *gdf6* 2	P3	GCA NON COD *sybu* 4	P5	GCA NON COD *sybu* 2
26	M5	CAT COD *gdf6* 6	P2	CAT COD *gdf6* 3	P4	CAT COD *gdf6* 1	P5	GCA NON COD *sybu* 3
27	M5	CAT transposon 1	P2	CAT COD *gdf6* 4	P4	CAT COD *gdf6* 2	P5	GCA NON COD *sybu* 4
28	M5	CAT transposon 2	P2	CAT COD *gdf6* 6	P4	CAT COD *gdf6* 3		
29	M5	CAT transposon 3	P2	CAT transposon 1	P4	CAT COD *gdf6* 4		
30	M5	CAT COD *sybu* 1	P2	CAT transposon 2	P4	CAT COD *gdf6* 6		
31	M5	TGC COD *sybu* 2	P2	CAT transposon 3	P4	CAT transposon 1		
32	M5	TGC COD *sybu* 3	P2	CAT COD *sybu* 1	P4	CAT transposon 2		
33	M5	TGC NON COD *gdf6* 1	P2	TGC COD *sybu* 2	P4	CAT transposon 3		
34	M5	TGC NON COD *gdf6* 2	P2	TGC COD *sybu* 3	P4	CAT COD *sybu* 1		

# - amplicon number per pool; n – sample identifier: female (F), male (M), phenofemale (P) 1-5; Oligonucleotides f/r see Supplementary Table 8.

SUPPLEMENTARY METHODS

Tol2 transgenesis

The plasmid pDTactb2:gdf6Y, which contains the *D. rerio actb2*-promoter driven *gdf6Y* expression cassette, was generated by combining the Tol2kit[4] vectors #395 (pDestTol2CG2) and #299 (p5E-*bactin2*) with pMEgdf6Y and p3Egdf6YpA in a Gateway LR Clonase II (Thermo Fisher Scientific) reaction according to the manufacturer's instructions. To generate plasmids pMEgdf6Y and p3Egdf6YpA, the *gdf6Y* genomic sequence from start- to stop-codon (including the intron) or the 965 bp sequence immediately downstream of *gdf6Y*, respectively, were amplified using Phusion High-Fidelity Polymerase (Thermo Fisher Scientific) according to the manufacturer's instructions. The applied oligonucleotides added the respective *att*-sites to recombine the obtained amplicons into pDONR221 (Thermo Fisher Scientific) or pDONRP2R-P3 (Tol2kit #220) for the generation of pMEgdf6Y or p3Egdf6YpA, respectively, using Gateway BP Clonase II (Thermo Fisher Scientific) according to the manufacturer's instructions. *Tol2*-mRNA was *in vitro* transcribed from the *NotI*-linearized Tol2kit[4] vector #396 (pCS2FA-*transposase*) using the mMessage mMachine SP6 Kit (Thermo Fisher Scientific) according to the manufacturer's instructions and quality-controlled by RNA agarose gel electrophoresis. The injection solution contained 20 ng/µl of pDTactb2:gdf6Y, 10 ng/µl of *Tol2*-mRNA, 30 ng/µl of *GFP*-mRNA, and 0.1 % phenol red[5].

Estimation of mutation rates

To determine mutation rates of *gdf6Y* and *gdf6X* in mosaic XY* animals, the pan-*gdf6*-PCR was performed as described or using Phusion High-Fidelity Polymerase (Thermo Fisher Scientific)

according to the manufacturer's instructions. The obtained DreamTaq and Phusion amplified fragments were cloned into pCRII-TOPO using the TOPO TA Cloning Kit (Thermo Fisher Scientific) according to the manufacturer's instructions or the pUC57-derivative pGGC[6] via *SmaI* cut-ligation, respectively. Individual clones were Sanger sequenced with M13F in-house by the FLI Core Facility Next Generation Sequencing or externally by Eurofins Genomics TubeSeq Service. Pan-*gdf6*-PCR amplicons from XX animals, injected with the *gdf6Y* sgRNAs Y1 and Y2, were purified from the reaction with NucleoSpin Gel and PCR Clean-up Mini kit (Macherey-Nagel) according to the manufacturer's instructions and subjected to a T7 Endonuclease assay (T7EI) and Sanger sequencing with gdf6-gtF2 by Eurofins Genomics TubeSeq Service. For the T7EI assay, 200 ng of purified PCR product and 1 µl of NEB 2 Buffer (New England Biolabs) in 9.5 µl total volume were first denatured for 5 min at 95°C and then rehybridized by cooling the sample at a rate of 2°C/s to 85°C and a rate of 0.1°C to 25°C to obtain potential heteroduplex DNA. To detect potential heteroduplex DNA by cleavage, 0.5 µl of T7EI (New England Biolabs) were applied for 15 min at 37°C and the reaction was analyzed by agarose gel electrophoresis. Sanger sequences from individual clones and PCR products were analyzed with Geneious Prime 2019.2.3.

To determine *gdf6X* and *gdf6Y* mutation rates in mosaic animals, injected with the *gdf6X* sgRNAs X1 and X2, the *gdf6Y*-specific PCR was performed on X*Y, and the pan-*gdf6*-PCR was performed on X*Y and X*X* individuals. PCR products were Sanger sequenced with the forward primer of the respective PCR by Eurofins Genomics TubeSeq Service. Sequences of the *gdf6Y*-specific PCR on X*Y and the pan-*gdf6*-PCR on X*X* animals were analyzed with Synthego Performance Analysis, ICE Analysis. 2019. v3.0. Synthego; [accessed between 03/05/2021 and 03/19/2021] by supplying the sgRNA Y2 or both *gdf6X*-sgRNA sequences, respectively. Sequences of the pan-

gdf6-PCR on X*Y animals were analyzed with Synthego Performance Analysis, ICE Analysis. 2019. v3.0. Synthego; [accessed between 03/05/2021 and 03/19/2021] by supplying both *gdf6X*-sgRNA sequences and the *gdf6Y*-sequence as a donor sequence to exclude the *gdf6Y* wild-type sequence from analysis of the *gdf6X* mutation rate. The result was evaluated by calculating the mutation rate as the percentage proportion of predicted indel sequences from the sum of indel and wild-type sequences.

Respective PCR products from individual mosaic animals for *foxl2l*, *clybl*, *id1*, and *zfp36l2* were first examined for large indel mutations. Those samples without obvious indels were subjected to a T7EI assay as described above. T7EI-resistant samples were declared not mutated, while cleaved PCR products from *foxl2l* samples were Sanger sequenced with foxl2lt2F by Eurofins Genomics TubeSeq Service. *Foxl2l*-Sequences were analyzed with Synthego Performance Analysis, ICE Analysis. 2019. v3.0. Synthego; [accessed on 11/17/2021 and 11/23/2021] by supplying the two *foxl2l*-sgRNA sequences and evaluated as described above. This strategy was also applied to exemplary *clybl*, *id1*, and *zfp36l2* PCR products accessing the Synthego Performance Analysis, ICE Analysis. 2019. v3.0. Synthego; on 11/24/2023-04/08/2024, 03/19/2024, and 11/22/2024, respectively.

Whole mount *in situ* hybridization (WISH)

Probes were prepared using the oligonucleotides presented in Supplementary Table 7. The digoxigenin-labeled antisense and sense *sybu* riboprobes were synthesized from a *SacII*- or *SacI*-linearized pGEM-T easy (Promega), which contained an 823 bp cDNA-fragment from *sybu*-mRNA (NCBI Reference Sequence: XM_015949383.2), using the plasmids SP6 or T7 promoter, respectively. The three *gdf6X*-CDS probe fragments were amplified using DreamTaq DNA Polymerase (Thermo Fisher Scientific) from the genomic DNA of an *N. furzeri* GRZ female with

the respective oligonucleotides and cloned using the TA Cloning™ Kit, Dual Promoter, with pCR™II Vector (Invitrogen) according to the manufacturer's instructions. The three antisense and sense *gdf6X* riboprobes were synthesized from purified *SpeI*- or *EcoRV*-digested M13-PCR products of the respective plasmid clone with a T7 or SP6 polymerase (ROCHE), respectively, according to the manufacturer's instructions. 500 ng/ml of each probe was applied in the respective sense or antisense probe mix.

Specimens were fixed in 4% paraformaldehyde/PBS overnight at 4°C and processed for whole mount *in situ* hybridization as described previously[7]. Before probe hybridization overnight at 65°C, samples were bleached in 6 % hydrogen peroxide/PBST for 1 h at room temperature, followed by a 30 or 6 min 5 µg/ml Proteinase K-treatment of the 0 dph or 11 dpf samples, respectively. After probe detection, hatchlings were again fixed in 4% paraformaldehyde/PBS, taken through a methanol series, equilibrated, and oriented for imaging in a clearing solution (1/3 benzyl benzoate, 2/3 benzyl alcohol). Images were acquired using the Zeiss SteREO Discovery.V8 equipped with a Zeiss AxioCam MRc (0 dph samples) or with a Zeiss Axio Zoom.V16 equipped with a Zeiss AxioCam HRc and an HXP 200 C light source (11 dpf samples). Images were processed in ZEN 2.6 (Blue Edition, Zeiss). The sex of MZM0403 hatchlings was subsequently determined by sequencing PCR-amplicons (Supplementary Table 4).

Bisulfite sequencing

Genomic DNA from testes or ovaries was isolated using the QIAamp DNA Mini Kit (QIAGEN) according to the protocol of the manufacturer. The deamination was performed with the EZ DNA Methylation-Gold Kit (Zymo Research) according to the manufacturer's instructions. After deamination, samples were stored at -20°C. PCRs with the designated oligonucleotides

(Supplementary Table 8) were performed to amplify the region of interest. The reaction mix was composed of 10 µl MyTaq HS Red Mix (Meridian Bioscience), 2 µl deaminated DNA, and the respective oligonucleotides at a concentration of 0.5 µM each in 20 µl total volume, and amplified using the following program: initial denaturation at 95°C for 3 min, 30 cycles of denaturation at 95°C for 30 s, annealing at 55°C for 30s, and elongation at 72°C for 1 min, followed by a final elongation at 72°C for 5 min. Amplicons were quality-controlled by agarose gel electrophoresis and distributed in equal amounts into 8 pools. Amplicons per pool were tagged per individual and locus, see Supplementary Table 9. The pools of amplicons were purified using Agencourt AMPure XP magnetic beads (Beckman Coulter) according to the manufacturer's instructions. Amplicon pools were quality-checked using the 2100 Bioanalyzer instrument in combination with the DNA 7500 assay (both Agilent Technologies). An individually tagged library was prepared from 125 ng of input material for each pool using the TruSeq DNA PCR-Free kit (Illumina). Since pools of amplicons do not need to be fragmented, the protocol was started with the "Repair Ends" step. The quantification and quality-check of libraries were done as described above. Libraries were pooled and sequenced on the MiSeq system. It ran in 151 cycle/paired-end mode (MiSeq reagent kit v2 - 300 cycles). Sequence information was converted to FASTQ format using bcl2fastq v2.20.0.422.

All the paired-end sequencing fastq-files were merged using clc_overlap_reads (CLC-Workbench, QIAGEN), as our in-house scripts for methylation rate calculation[8] require single-end reads. In the first step, amplicons contained in a pool (`amplicons.fq`) were separated by providing the relevant information via the file pool_composition.csv to the script `pool_separator.py` (see Supplementary Table 9 for pool information). The .csv-file contains semicolon-separated information for each amplicon of the pool (i.e., pool; 3 bp tag; forward primer; reverse primer; sample name). The command to run `pool_separator.py` is as follows:

```
python3 pool_separator.py amplicons.fq pool_composition.csv
```

The in-house script `methi.py` was used to calculate the X and Y chromosome-specific CpG methylation rates, for predefined CpGs within the amplicons. The script requires information about the primer (Supplementary Table 8), the chromosome-specific length of the amplicons, the tag length, the positions of all CpGs within the amplicon (comma-separated), a position of a non-CpG cytosine (cx) as well as signature sequences that are unique for the respective chromosome X (p1) and Y (p2). This information was provided to the tool via a text file, as shown below, exemplified for COD_gdf6_1:

```
forward_primer:ATTTAAAAGTTGTTGGAATAAATTTTATTT
reverse_primer:TTATTAACTAATACACCAAAATTAAAAAAC
expected_length_p1:132
expected_length_p2:134
tag_length:3
cpg_pos_p1:47,59,64,79
cpg_pos_p2:50,61,66,81
cx_pos_p1:53
cx_pos_p2:56
p1:TGTAATATAA
p2:GTAAAAGTATA
```

The command to run `methi.py` is shown below:

```
python3 methi.py -d fastq_files/ -i info.txt -s
```

whereby -s turns on the chromosome-wise calculation of the methylation rate. The script also returns the methylation rate of the cytosine that is not in a CpG-context (non-conversion rate [*r*]), which is used to correct the methylation rates of the CpGs (CpG_{corr}). The median of all non-

conversion rates across all samples is calculated (r_{all}) and used to correct the CpG methylation rate (CpG) with equation (3):

(3) $$CpG_{corr} = \frac{CpG - r_{all}}{1 - r_{all}}$$

Alternatively, the methylation rate of a CpG was determined sample-wise with Geneious Prime 2019.2.3 by assembling the pool-separated reads to a bisulfite-conversed reference sequence of the respective amplicon and measuring the proportion of cytosines at the given position. The obtained methylation rate was corrected with the formula above.

The corrected methylation rates of all analyzed CpGs and samples were visualized using Morpheus [https://software.broadinstitute.org/morpheus].

SUPPLEMENTARY REFERENCES

1. Reichwald K*, et al.* Insights into Sex Chromosome Evolution and Aging from the Genome of a Short-Lived Fish. *Cell* **163**, 1527-1538 (2015).

2. Richter A, Krug J, Englert C. Molecular Sexing of Nothobranchius furzeri Embryos and Larvae. *Cold Spring Harb Protoc* **2022**, 630-640 (2022).

3. Racz GA, Nagy N, Tovari J, Apati A, Vertessy BG. Identification of new reference genes with stable expression patterns for gene expression studies using human cancer and normal cell lines. *Sci Rep* **11**, 19459 (2021).

4. Kwan KM*, et al.* The Tol2kit: a multisite gateway-based construction kit for Tol2 transposon transgenesis constructs. *Developmental dynamics : an official publication of the American Association of Anatomists* **236**, 3088-3099 (2007).

5. Hartmann N, Englert C. A microinjection protocol for the generation of transgenic killifish (Species: Nothobranchius furzeri). *Developmental dynamics : an official publication of the American Association of Anatomists* **241**, 1133-1141 (2012).

6. Geissler R*, et al.* Transcriptional activators of human genes with programmable DNA-specificity. *PloS one* **6**, e19509 (2011).

7. Hauptmann G, Gerster T. Two-color whole-mount in situ hybridization to vertebrate and Drosophila embryos. *Trends in genetics : TIG* **10**, 266 (1994).

8. Schwarz R. The master male sex determinant Gdf6Y of the turquoise killifish arose through allelic neofunctionalization. *Zenodo*, https://doi.org/10.5281/zenodo.14186262 (2024).
