# Supplementary material for: The master male sex determinant Gdf6Y of the turquoise killifish arose through allelic neofunctionalization
Source: Nat Commun. 2025 Jan 9;16:540. doi: 10.1038/s41467-025-55899-7 (PMC11718055; doi:10.1038/s41467-025-55899-7)
Supplement: Supplementary file 3 — Description of Additional Supplementary Files [file 41467_2025_55899_MOESM3_ESM.pdf]

Description of Additional Supplementary Files

File Name: Supplementary Data 1
Description: Analyses results from previously published RNA-Seq data (Reichwald et al. Cell 2015) of female and male N. furzeri samples at different ages.

File Name: Supplementary Data 2
Description: RNA-Seq data analyses results from TM4 cells transfected with a gdf6Y or control expression vectors.

File Name: Supplementary Data 3
Description: RNA-Seq data analyses results from HeLa cells transfected with a gdf6Y or control expression vectors.
