# Supplementary material for: The master male sex determinant Gdf6Y of the turquoise killifish arose through allelic neofunctionalization
Source: Nat Commun. 2025 Jan 9;16:540. doi: 10.1038/s41467-025-55899-7 (PMC11718055; doi:10.1038/s41467-025-55899-7)
Supplement: Supplementary file 7 — Reporting Summary [file 41467_2025_55899_MOESM7_ESM.pdf]

nature portfolio

Corresponding author(s): Annekatrin Richter
Christoph Englert

Last updated by author(s): Dec 12, 2024

Reporting Summary

Nature Portfolio wishes to improve the reproducibility of the work that we publish. This form provides structure for consistency and transparency in reporting. For further information on Nature Portfolio policies, see our Editorial Policies and the Editorial Policy Checklist.

Statistics

For all statistical analyses, confirm that the following items are present in the figure legend, table legend, main text, or Methods section.

n/a	Confirmed	
☐	☒	The exact sample size (*n*) for each experimental group/condition, given as a discrete number and unit of measurement
☐	☒	A statement on whether measurements were taken from distinct samples or whether the same sample was measured repeatedly
☐	☒	The statistical test(s) used AND whether they are one- or two-sided<br>*Only common tests should be described solely by name; describe more complex techniques in the Methods section.*
☒	☐	A description of all covariates tested
☐	☒	A description of any assumptions or corrections, such as tests of normality and adjustment for multiple comparisons
☐	☒	A full description of the statistical parameters including central tendency (e.g. means) or other basic estimates (e.g. regression coefficient) AND variation (e.g. standard deviation) or associated estimates of uncertainty (e.g. confidence intervals)
☐	☒	For null hypothesis testing, the test statistic (e.g. *F*, *t*, *r*) with confidence intervals, effect sizes, degrees of freedom and *P* value noted<br>*Give P values as exact values whenever suitable.*
☐	☒	For Bayesian analysis, information on the choice of priors and Markov chain Monte Carlo settings
☒	☐	For hierarchical and complex designs, identification of the appropriate level for tests and full reporting of outcomes
☒	☐	Estimates of effect sizes (e.g. Cohen's *d*, Pearson's *r*), indicating how they were calculated

Our web collection on statistics for biologists contains articles on many of the points above.

Software and code

Policy information about availability of computer code

Data collection	Microscopic data: ZEN 2.6 (Blue Edition, Zeiss)<br>Gel pictures: Bio-Rad Image Lab 6.0.1<br>Mutation rates: Synthego Performance Analysis, ICE Analysis. 2019. v3.0. Synthego<br>RT-qPCR data: Bio-Rad CFX Manager 3.1<br>Bisulfite sequencing, RNA-Seq data: bcl2fastq v2.20.0.422
Data analysis	Microscopic data: ZEN 2.6 (Blue Edition, Zeiss)<br>Image analysis and quantification: Arivis Vision4D (v4.1.2; now Zeiss Arivis)<br>Sequence analyses and sgRNA design: Geneious Prime 2019.2.3; CHOPCHOP v3<br>Mutation rates, RT-qPCR data, luciferase assay data: Microsoft Excel for Microsoft 365 MSO (Version 16.0.1.) 64-bit; GraphPad Prism version 9.0.2 for Windows, GraphPad Software, San Diego, California USA, www.graphpad.com<br>Bisulfite sequencing: clc_overlap_reads (CLC-Workbench, QIAGEN); pool_separator.py, methi.py [https://doi.org/10.5281/zenodo.14186262]; Geneious Prime 2019.2.3; Microsoft Excel for Microsoft 365 MSO (Version 16.0.1.) 64-bit; Morpheus [https://software.broadinstitute.org/morpheus]<br>RNA-Seq data: UMI-tools' extract command version 1.1.1; sga [https://github.com/jts/sga]; STAR 2.7.10a; FeatureCounts 2.0.3; R 4.1.3; R package GenomicAlignments; DESeq2 1.34.0; R scripts [https://doi.org/10.5281/zenodo.13790201]; Morpheus [https://software.broadinstitute.org/morpheus]; VolcaNoseR [https://huygens.science.uva.nl/VolcaNoseR2/]; WebGestalt 2019 [https://www.webgestalt.org/]

For manuscripts utilizing custom algorithms or software that are central to the research but not yet described in published literature, software must be made available to editors and reviewers. We strongly encourage code deposition in a community repository (e.g. GitHub). See the Nature Portfolio guidelines for submitting code & software for further information.

Data

Policy information about availability of data

All manuscripts must include a data availability statement. This statement should provide the following information, where applicable:

- Accession codes, unique identifiers, or web links for publicly available datasets
- A description of any restrictions on data availability
- For clinical datasets or third party data, please ensure that the statement adheres to our policy

The RNA-Seq data in this study have been deposited in NCBI's Gene Expression Omnibus under accession codes GSE263626 [https://www.ncbi.nlm.nih.gov/geo/query/acc.cgi?acc=GSE263626] (female and male N. furzeri samples at different life stages) and GSE233997 [https://www.ncbi.nlm.nih.gov/geo/query/acc.cgi?acc=GSE233997] (human HeLa and murine TM4 cells). RNA-Seq data analysis results generated in this study are provided as Supplementary Data 1, 2, and 3 for N. furzeri samples, TM4 cells, and HeLa cells, respectively. The bisulfite sequencing data in this study have been deposited in NCBI's Sequence Read Archive under accession code PRJNA974954 [https://www.ncbi.nlm.nih.gov/bioproject/?term=PRJNA974954]. Source data are provided with this paper.

Research involving human participants, their data, or biological material

Policy information about studies with human participants or human data. See also policy information about sex, gender (identity/presentation), and sexual orientation and race, ethnicity and racism.

Reporting on sex and gender	No research involving human participants, their data, or biological material was performed in this study.
Reporting on race, ethnicity, or other socially relevant groupings	No research involving human participants, their data, or biological material was performed in this study.
Population characteristics	No research involving human participants, their data, or biological material was performed in this study.
Recruitment	No research involving human participants, their data, or biological material was performed in this study.
Ethics oversight	No research involving human participants, their data, or biological material was performed in this study.

Note that full information on the approval of the study protocol must also be provided in the manuscript.

Field-specific reporting

Please select the one below that is the best fit for your research. If you are not sure, read the appropriate sections before making your selection.

☒ Life sciences ☐ Behavioural & social sciences ☐ Ecological, evolutionary & environmental sciences

For a reference copy of the document with all sections, see nature.com/documents/nr-reporting-summary-flat.pdf

Life sciences study design

All studies must disclose on these points even when the disclosure is negative.

Sample size	No statistical tests for sample size calculation were performed. To obtain statistically meaningful results, a sample size of at least 3 and ideally 5 or more was intended but in some cases restricted due to sample or data availability. To draw meaningful conclusions especially with lower sample numbers, experiments were at least performed twice (sequencing data) or validated using another experimental approach (validation of sequencing data with RT-qPCR).
Data exclusions	Two Mus musculus Sertoli-like TM4 cell samples (gdf6Y replicate 6 and gdf6Ydel9 replicate 6) were excluded from the DESeq2 analysis, because of lesser quality in the MultiQCv1.12 report (assigned reads by featureCounts < 20 M reads; FastQC Duplicate reads > 70 %) compared to all other samples (assigned reads by featureCounts > 20 M reads; FastQC Duplicate reads < 70 %).
Replication	Representative experiments were independently repeated twice with similar results.
Randomization	Randomization is not relevant to our study as experimental groups were determined by prior genotyping (animal experiments) or treatment (cell transfections).
Blinding	The investigators were not blinded to group allocation during data collection, because group allocation by genotyping (animal experiments) or treatment (cell transfections) were integral parts of the experimental procedures and performed by the investigators themselves. Blinding was not relevant to data analyses in this study due to the uninfluenceable nature of most experimental readouts (sequencing based experiments, phenotypic and microscopic analyses, luciferase reporter assay, RT-qPCR).

Reporting for specific materials, systems and methods

We require information from authors about some types of materials, experimental systems and methods used in many studies. Here, indicate whether each material, system or method listed is relevant to your study. If you are not sure if a list item applies to your research, read the appropriate section before selecting a response.

Materials & experimental systems

n/a	Involved in the study
☒	☐ Antibodies
☐	☒ Eukaryotic cell lines
☒	☐ Palaeontology and archaeology
☐	☒ Animals and other organisms
☒	☐ Clinical data
☒	☐ Dual use research of concern
☒	☐ Plants

Methods

n/a	Involved in the study
☒	☐ ChIP-seq
☒	☐ Flow cytometry
☒	☐ MRI-based neuroimaging

Eukaryotic cell lines

Policy information about cell lines and Sex and Gender in Research

Cell line source(s)	HeLa, female (Human cervix carcinoma cells), DSMZ no.: ACC 57, Leibniz Institute DSMZ - German Collection of Microorganisms and Cell Cultures GmbH, Inhoffenstrasse 7B, 38124 Braunschweig, GERMANY<br>TM4, male (Murine Sertoli cells), Cat. No. 88111401, The European Collection of Authenticated Cell Cultures (ECACC), UK Health Security Agency, Porton Down, Salisbury, SP4 0JG, UK<br>HEK293, female (human embryonic kidney cells transformed by adenovirus type 5), DSMZ no.: ACC 305, Leibniz Institute DSMZ - German Collection of Microorganisms and Cell Cultures GmbH, Inhoffenstrasse 7B, 38124 Braunschweig, GERMANY<br>OLF-136, male (Oryzias latipes/medaka fibroblast cells), Cell No. RCB0184, Cell Bank, RIKEN BioResource Research Center (BRC) 3-1-1 Koyadai, Tsukuba, Ibaraki, 305-0074, Japan
Authentication	None of the cell lines used were authenticated by us.
Mycoplasma contamination	The cell lines are regularly tested for mycoplasma contamination and used only when negative.
Commonly misidentified lines<br>(See ICLAC register)	No commonly misidentified cell lines were used in this study as confirmed consulting the ICLAC register.

Animals and other research organisms

Policy information about studies involving animals; ARRIVE guidelines recommended for reporting animal research, and Sex and Gender in Research

Laboratory animals	GRZ: Nothobranchius furzeri laboratory wild-type strain collected in Zimbabwe in 1969 (Jubb R. A new Nothobranchius (Pisces, Cyprinodontidae) from Southeastern Rhodesia. Journal of the American Killifish Association 8, 12-19 (1971).)<br>MZM0403: Nothobranchius furzeri laboratory wild-type strain collected in Mozambique in 2004 (Terzibasi E, et al. Large differences in aging phenotype between strains of the short-lived annual fish Nothobranchius furzeri. PloS one 3, e3866 (2008).)<br>Genetically modified Nothobranchius furzeri in GRZ background were generated in this study at the Leibniz Institute on Aging – Fritz Lipmann Institute Jena, Germany. Generated gdf6Y-inactivation lines with identical phenotype (male-to-female sex reversal): GRZ-gdf6Ydel9; GRZ-gdf6Ydel113; GRZ-gdf6Ydel6,del8 (discontinued). Generated gdf6X-inactivation line: GRZ-gdf6Xdel113.<br>Substrain, genetic background, and age in Fig. 1b,c: gdf6Y-inactivated F0 CRISPants and wild-type GRZ, 3 months; 1d,f,g: gdf6Y-inactivated GRZ F1 generation, 1d: 1 month, 1f,g: 2.4 months; 1h: GRZ-gdf6Ydel9, 17-24 days post fertilization (dpf); 1i RL: GRZ-gdf6Ydel6,del8, BF: GRZ-gdf6Ydel9, 0 days post hatching (dph); 2c-h: GRZ-gdf6Xdel113, 2c: 1 month, 2d: 0 dph, 2e-h: 2 months; 3a-c: GRZ, 3a: 3 months, 3b-c: 0 dph; 4a-c: GRZ, 10 dpf, 0 dph, 3 dph; 4e: foxl2l-CRISPants in GRZ-gdf6Ydel9 background, 0 dph; 5b-f: gdf6Y-BAC transgenic F0 and wild-type GRZ, 4.2 months; 6d: GRZ, 10 dpf (id1), 3 months (zfp36l2); 6e: id1-CRISPants in GRZ background; 6f: zfp36l2-CRISPants in GRZ background; 6e,f: 0 dph<br>Substrain, genetic background, and age in Supplementary Fig. 2a: gdf6Y-inactivated F1 GRZ individual, 2.4 months; 2b: GRZ-gdf6Ydel9, 1.5 months; 2c,e,g: GRZ-gdf6Ydel113; 2d,f,h: GRZ-gdf6Ydel9; 2c-h: 3 months; 3a,c: GRZ-gdf6Ydel6,del8, 19-26 dpf; 3b: GRZ-gdf6Ydel113, 0dph; 3d: GRZ-gdf6Ydel9, 0 dph; 3f: gdf6X-inactivated F0 CRISPants, 1 and 23 dph; 3g: gdf6X-inactivated F1 generation in GRZ-gdf6Ydel9 background, 0 dph; 3h-j: GRZ-gdf6Xdel113; 3h: 0 dph; 3i: 3, 7, and 21 dph; 3j: 2 months; 4a: GRZ, 4 months; 4b: GRZ-gdf6Ydel113, 3 months; 4c-h: GRZ; 4c,d: 3 months; 4e-g: 0 dph; 4h: 10 dpf, 0 dph, 3 dph, 3 months; 4i: MZM0403, 0 dph; 5c: GRZ-gdf6Ydel113, 2 months; 5d: gdf6Y-inactivated GRZ F1 generation, 2.4 months; 6a: GRZ-gdf6Ydel113, 0 dph; 6b-g: GRZ; 6b: 0 dph, 3 dph; 6c-d: 0 dph; 6e: 3 dph; 6f,g: 7-14 dpf; 6h: GRZ-gdf6Ydel9, 11 dpf; 7a-b: GRZ, 10 dpf, 0 dph, 3 dph, 3 months; 7c: GRZ-gdf6Ydel113, 0 dph, gdf6Y-inactivated GRZ F1 generation, 2.4 months; 7d,e: GRZ, 0 dph; 7f: GRZ, 5 weeks; 7g-i: foxl2l-CRISPants in GRZ-gdf6Ydel9 background, 0 dph; 7k,l,m: gdf6Y Tol2-transgenic F0 in GRZ background; 7k,l: 0 dph; 7m: 2 dph; 8d: GRZ, 10 dpf, 0 dph, 3 dph, 3 months; 8e: GRZ-gdf6Ydel9, GRZ-gdf6Ydel113, 3 months; 9a: GRZ, 0 dph, 3.5 months; 9b: id1-CRISPants in GRZ background; 9c: zfp36l2-CRISPants in GRZ background; 9b,c: 0 dph
Wild animals	The study did not involve wild animals.
Reporting on sex	Sex or sexual differences are the main subject of this study. Hence, sex was considered in the study design and analysis. Genetic sex is the major determiner of the experimental groups used in this study and was assigned by molecular identification of the sex chromosomes within the individual samples. Markers of the phenotypic sex/sexual differences are the major readout in most

	experiments presented in this study. Therefore, all experiments are reported disaggregated for sex.
Field-collected samples	This study did not involve samples collected in the field.
Ethics oversight	All animals were maintained in the fish facility of the Leibniz Institute on Aging – Fritz Lipmann Institute Jena according to the German Animal Welfare Law. All experiments were covered by animal experiment licenses 003-005/12, 03-044/16, FLI-18-020, and FLI-21-012 approved by the Thuringian authorities (Thüringer Landesamt für Verbraucherschutz).

Note that full information on the approval of the study protocol must also be provided in the manuscript.
